# Bacterial Diversity and Community Structure in Korean Ginseng Field Soil Are Shifted by Cultivation Time

**DOI:** 10.1371/journal.pone.0155055

**Published:** 2016-05-17

**Authors:** Ngoc-Lan Nguyen, Yeon-Ju Kim, Van-An Hoang, Sathiyamoorthy Subramaniyam, Jong-Pyo Kang, Chang Ho Kang, Deok-Chun Yang

**Affiliations:** 1 Korean Ginseng Center and Ginseng Genetic Resource Bank, Kyung-Hee University, Yongin-si, Gyeonggi-do, Republic of Korea; 2 Plant Molecular Biology and Biotechnology Research Center, Gyeongsang National University, JinJu-si, Gyeongsangnam-do, Republic of Korea; 3 Graduation of Biotechnology, Kyung-Hee University, Yongin-si, Gyeonggi-do, Republic of Korea; Chengdu Institute of Biology, CHINA

## Abstract

Traditional molecular methods have been used to examine bacterial communities in ginseng-cultivated soil samples in a time-dependent manner. Despite these efforts, our understanding of the bacterial community is still inadequate. Therefore, in this study, a high-throughput sequencing approach was employed to investigate bacterial diversity in various ginseng field soil samples over cultivation times of 2, 4, and 6 years in the first and second rounds of cultivation. We used non-cultivated soil samples to perform a comparative study. Moreover, this study assessed changes in the bacterial community associated with soil depth and the health state of the ginseng. Bacterial richness decreased through years of cultivation. This study detected differences in relative abundance of bacterial populations between the first and second rounds of cultivation, years of cultivation, and health states of ginseng. These bacterial populations were mainly distributed in the classes Acidobacteria, Alphaproteobacteria, Deltaproteobacteria, Gammaproteobacteria, and Sphingobacteria. In addition, we found that pH, available phosphorus, and exchangeable Ca^+^ seemed to have high correlations with bacterial class in ginseng cultivated soil.

## Introduction

In East Asian traditional medicine, *Panax ginseng* (Korean ginseng) has been considered to be an “adaptogen” for thousands of years. Korean ginseng displays significantly potent pharmacological activities, such as enhancing immune system function, central nervous system activities, and physical and sexual functions. More specifically, these activities include anti-stress, anti-aging, anti-fatigue, anti-oxidative, anti-diabetes, anti-cancer [1], anti-atopic, and anti-inflammatory actions [2], benefiting liver function and preventing liver disease [3,4], and preventing osteoporosis [5]. Korea has become one of the leading producers of ginseng worldwide, as described in a previous study [6]. The production of harvestable ginseng roots requires a cultivation period, which is the time required for ginseng roots to reach maturity, of 4 to 6 years. The use of long-term ginseng monoculture may lead to changes in the bacterial community in ginseng-cultivated soil. Ginseng is also known to become susceptible to disease after 4 years of cultivation, as described by Ohh et al. [7]. In Korea, disease in ginseng has been demonstrated to cause yield losses of up to 30%-60% [8], and more severe diseases, caused either by physiological disorders or infectious microorganisms, are known to appear in replanted ginseng crops [9]. This crop failure can occur during the second round of cultivation, even if fertilizers are supplied after harvest to restore soil nutrient balance. Therefore, the microorganisms resident in these soil samples are of great importance because they perform vital processes such as decomposition, mineralization, aeration, and recycling. Cumulatively, these processes increase the amount of available nutrients for plants, increase the extent of soil aggregation, and are important for suppressing disease. To date, several studies have attempted to investigate the resident bacterial communities in ginseng soil. For example, rhizobacteria belonging to the phyla Actinobacteria and Firmicutes were isolated from 1- to 3-year-old ginseng rhizosphere soil samples using a culture-dependent method [10]. However, small factions of bacterial populations exist in soil can be cultured in laboratory. Traditional molecular methods, such as amplified polymorphic DNA [11,12] and denaturing gradient gel electrophoresis [13], have also been extensively used to study the bacterial and fungal populations in ginseng soil. Despite the use of these methods, only certain predominant microbial groups can be detected. Therefore, the full extent of microorganism diversity in ginseng soil is still poorly understood. Recent advances in the 454 pyrosequencing method have shown great promise in increasing our understanding of the extent of bacterial diversity in soil [14–18].

Therefore, this study investigated the bacterial diversity, community structure and core bacterial populations in various Korean ginseng soil samples *via* amplification of the bacterial 16S rRNA gene V1-V3 region and analyzed the amplification products using the 454 GS FLX Titanium platform (454 Life Science, Rosche) in Chunlab, Inc. (Korea). Our goals were to construct a comprehensive summary of bacterial diversity and community structure in Korean ginseng cultivated soil and to investigate changes in the bacterial population over multiple years of cultivation (0, 2, 4, and 6 years of cultivation), multiple rounds of cultivation (non-cultivated, first round, and second round), different soil depths (0–10 cm, 10–20 cm, and 20–30 cm), and different health states of ginseng (healthy and unhealthy).

We hypothesized that the soil bacterial reservoir would become less diverse over time. Since ginseng roots grow to depths of approximately 10–25 cm, the exudates of those roots may affect the surrounding soil bacterial population. Consequently, we hypothesized that the bacterial community would be different at different soil depths. Finally, it was hypothesized that the microbial taxonomy would be different in healthy and unhealthy ginseng soil samples.

## Materials and Methods

Field sampling and molecular research permits were granted by Gyeonggi do Agricultural Research & Extension Services (project no. PJ008813042014). All necessary permits were obtained for the field study, and the study did not involve endangered or protected species. No other permissions were required.

### Site description and soil sampling

In April 2012, 30 soil samples were collected from different ginseng cultivation areas in the northern part of South Korea. Information of soil is shown in S1 Table. In these areas, the famers cultivated the same commercialized *Panax ginseng* seeds. During ginseng cultivation, artificial fertilizers and pesticides were not apply to soil. Soil samples were collected in the first round of ginseng cultivation at years 2, 4, and 6 and in the second round of cultivation in years 2, 4, and 6 (designated R2, R4, and R6, respectively). The soil samples without ginseng cultivation before, but prepared for ginseng cultivation, were collected for using as control or year 0 of cultivation. After removing organic debris from the soil surface, samples were collected from depths of 0–10, 10–20, and 20–30 cm. The ginseng plants with dark green leaves, normal stem, light colored fleshy root, and no lesion on the surface of root, were considered as healthy ginseng (A). The ginseng plants with disease phenomenon such as wilt stem, not dark green colored leaves, root rots, and black holes on the surface, were considered as unhealthy (B). The soil samples taken from healthy (A) and unhealthy (B) ginseng plants were responded as healthy (A) and unhealthy (B), respectively. The 3×3 m^2^ subplots were collected, and 10 samples from each site were pooled. Soil samples were kept in Ziploc bags in icebox and then transferred to the laboratory, where they were stored in -50°C until further analysis.

### Analysis of soil characteristics

Soil samples were air-dried and passed through a 2-mm sieve. Selected chemical properties of these samples were analyzed according to the standard methods of the Rural Development Administration, South Korea [19]. Soil pH and electrical conductivity (EC) were measured with a pH meter (Thermo, Orion 900A) and an Orion 162A conductivity meter, respectively, at a soil-to-water ratio of 1:5. Soil organic matter (OM) content, phosphorus content, and the levels of available cations (K^+^, Ca^2+^, Mg^2+^, and Na^+^) were assessed using the Tyurin method, the Lancaster method, and the 1N-NH_4_OAc (pH 7.0) method, respectively. The amount of NO_3_-N was determined using an automatic wet chemical analyzer (Bran Luebbe- AA3).

### DNA extraction and pyrosequencing

DNA was extracted using a Soil DNA Isolation Kit (MO BIO Laboratories, CA, USA) according to the manufacturer’s instructions. Extracted DNA was stored at -20°C until analysis. The V1-V3 region of the bacterial 16S rRNA gene was amplified from each sample using the 27F and 518R primers. Fusion primers included the 454 pyrosequencing adapters, keys, linkers, and barcodes, with the latter present only in the 518R-fusion primers. PCR reactions were carried out as previously described [20]. DNA sequencing was performed by ChunLab, Inc. (Seoul, South Korea) using a Roche/454 GS FLX Titanium platform, according to the manufacturer’s instructions.

### Processing of sequencing data and taxonomic analysis

Sequences were processed and analyzed according to the bioinformatics procedures described by Chun et al. [21] and Singh et al. [22]. Raw sequencing reads from different soil samples were separated by unique barcode sequences. Sequences with short lengths (<300 bp) or including >2 ambiguous bases (Ns) were removed before analysis. Primer, linker, and barcode sites were then trimmed by pairwise alignment. Nonspecific PCR amplicons that showed no matches in the 16S rRNA gene database using either the Hidden Markov Model or the EzTaxon database were discarded. All sequence reads were additionally screened for chimeras using the BLAST program. Taxonomic assignment was carried out by comparing the sequence reads against the EzTaxon-e database, using a combination of the initial BLAST-based searches and additional pairwise similarity comparisons. The following criteria were applied for the taxonomic assignment of each read (x = distance values): species (x≤0.03), genus (0.03<x≤0.05), family (0.05<x≤0.1), order (0.1<x≤0.15), class (0.15<x≤0.2), and phylum (0.2<x≤0.25) [23–26]. If the distance was greater than the cutoff value, the read was assigned to an unclassified group. If the sequence cluster could not be identified with a valid name, the accession number of the GenBank sequence entry sharing the highest sequence similarity with the sequence cluster was used as a provisional name.

The sequences obtained in this study have been deposited into the NCBI short-read archive database under accession number SRP047259.

### Statistical analysis

Operational taxonomic units (OTUs), defined as units with 97% sequence similarity, were used to generate rarefaction curves and to estimate the richness of these units using the abundance-based coverage estimate (ACE), Chao1, and Shannon non-parametric indices using the MOTHUR program [27]. The un-weighted pair group method with the arithmetic mean (UPGMA) was also performed on the weighted-normalized UniFrac calculation [28]. These analyses were carried out using the CLcommunity software (ChunLab Inc.). Comparative richness and diversity indices were determined using the Tukey *post hoc* test, in ANOVA (XLSTAT). Non-metric multidimensional scaling (NMDS) ordination, permutational analysis of variance (PERMANOVA), and similarity percentage (SIMPER) tests for significant differences in bacterial community structure were conducted using the Past3 software [29] based on the Bray-Curtis matrix of relative abundance data. Boxplots showed significant differences in relative abundances of bacterial populations between rounds of cultivation were also conducted using the Past3 software [29]. Heat maps of core bacterial classes and families were generated using the MeV software version 4.9.0 [30]. A network of correlations of soil chemical compositions and core classes was constructed using the Cytoscape software version 3.2.1 [31]. Bubble charts and other statistical analyses were performed in the R2.15.3 software. All statistical tests were considered significant at *p*<0.05.

## Results

### Soil edaphic properties

The soil chemical compositions of the soil samples are shown in S1 Fig and S2 Table. The relationships between pH and all of the measured variables are shown in S1 Fig (first row and column). The data in S1 Fig indicates that the amounts of phosphorus (P_2_O_5_) and exchangeable Ca^2+^ vary according to soil pH. After planting, soil pH and level of exchangeable Ca^2+^ decreased, albeit to varying extents, in the second round of cultivation. The levels of exchangeable Na^+^ and available NO_3_-N exhibited strong positive correlations with electrical conductivity (EC). The amounts of organic matter (OM) and available P_2_O_5_ were also positively correlated with each other and negatively correlated with the level of exchangeable Mg^2+^. In addition, the levels of exchangeable Ca^2+^ and exchangeable Mg^2+^ were also positively correlated with each other.

### Bacterial diversity indices are significantly different between 2- and 6-year-old ginseng soil samples

After quality filtering, trimming, and removal all chimeric reads, a total of 158,635 sequence reads of the bacterial 16S rRNA were obtained from 30 soil samples, with an average of 5,287 reads per sample. The OTUs ranged from 1,299 to 5,453.

**Fig 1:**
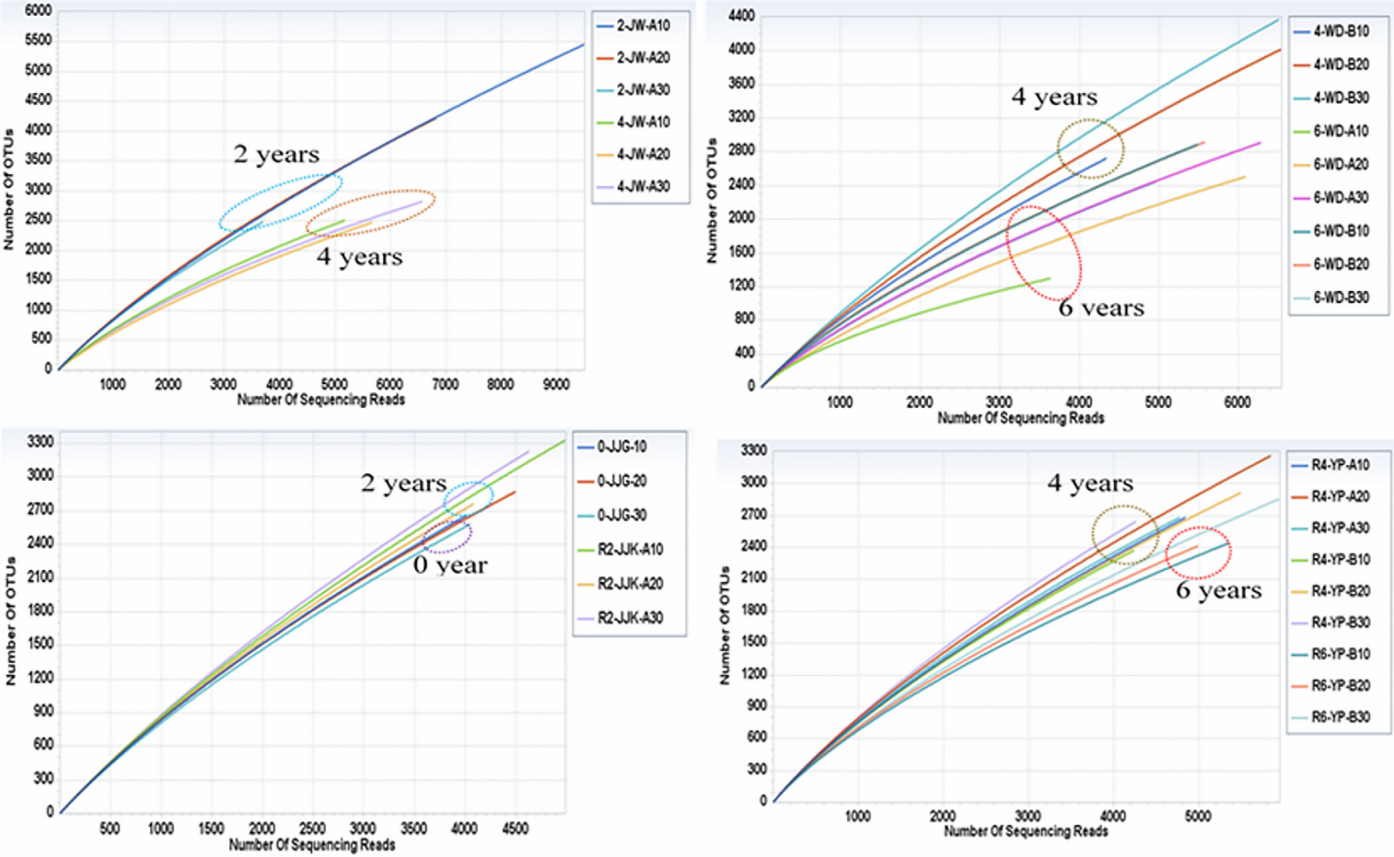
Rarefaction curves depicting the effect of sequencing on the number of operational taxonomic units (OTUs) within 30 soil samples. OTUs are shown at the 3% genetic distance levels.

Rarefaction curves (Fig 1) indicated that the number of detected OTUs increased with the number of sequences sampled in each of the soil samples. Moreover, none of the curves reached an asymptote. In the same sampling area, rarefaction curves may be shifted by years of ginseng cultivation, which increased more slowly than those in soil samples of long cultivation times.

**Fig 2:**
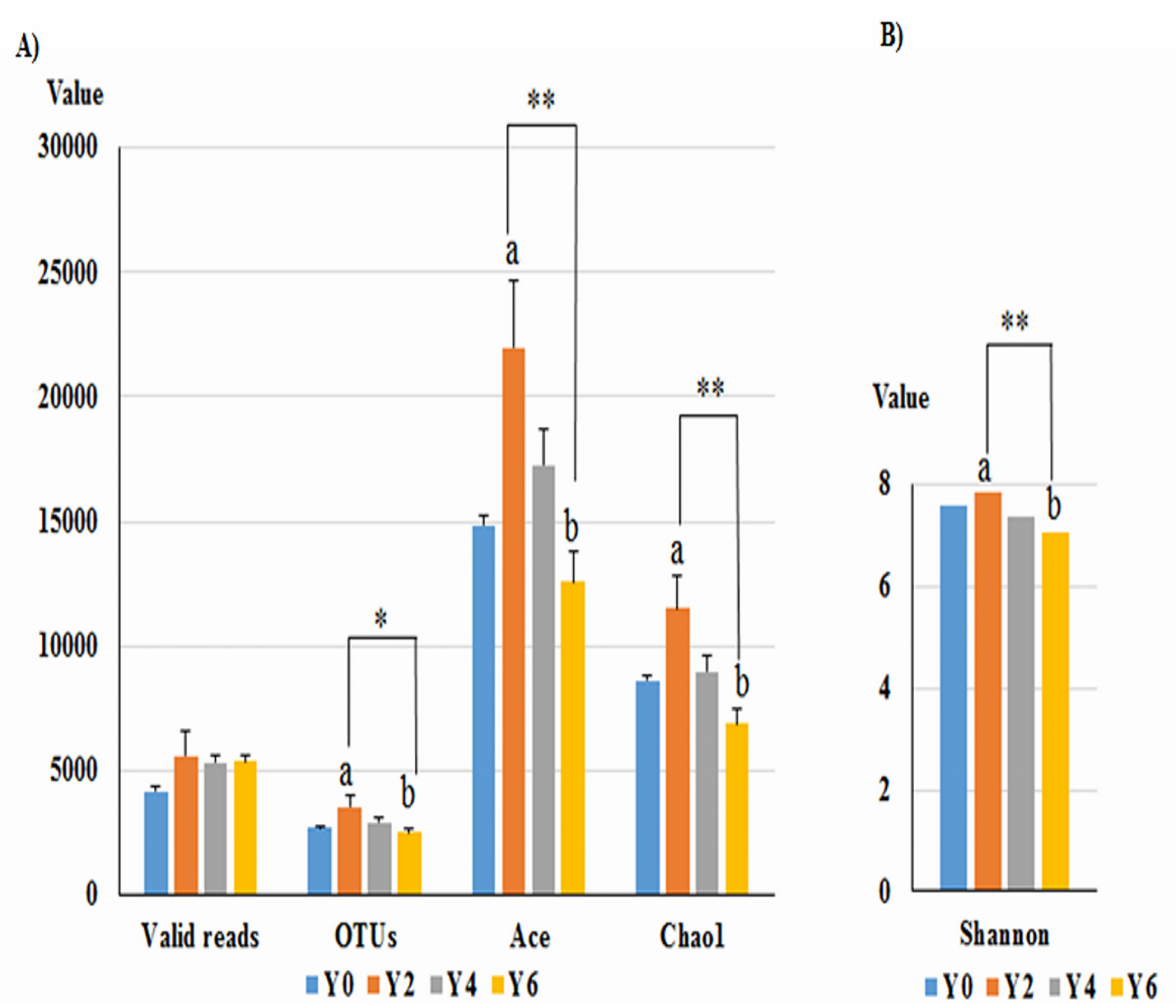
Richness and diversity indices along a time gradient. (A) Richness indices and (B) Shannon’s diversity index. 2-year-old (Y2, n = 6), 4-year-old (Y4, n = 12), 6-year-old (Y6, n = 9), and non-cultivated (Y0, n = 3) soil samples. All values are indicated as the mean ± SE. “a” and “b” indicate differences in alpha diversity indices values between 2-year-old and 6-year-old samples (one-way ANOVA by *post hoc* Tukey’s test). *, *p*<0.05; **, *p*<0.01.

The richness (OTUs) and diversity estimators (Ace, Chao1, and Shannon) are summarized in Table 1. The number of estimated OTUs was on average 2 to 3-fold greater than the number of observed OTUs. There were no significant differences in the richness and diversity estimators among geographic areas, soil depths, rounds of cultivation, or health states of ginseng (data not shown), which may demonstrate similar diversity between soil samples based on these factors. These indices (except valid reads) were able to demonstrate significant differences between 2-year-old (Y2) and 6-year-old (Y6) samples, with the Y2 samples showing the highest diversity and the Y6 samples showing the lowest diversity (*p*<0.01, Tukey’s test, Fig 2).

**Table 1:** Diversity indices obtained at a genetic distances of 3%.

Sample	Valid reads	OTUs	Ace	Chao1	Shannon
0-JJG-10	4009	2660	14647	8915	7.60
0-JJG-20	4489	2873	15478	8622	7.67
0-JJG-30	4043	2582	14470	8298	7.48
2-JW-A10	9471	5453	32110	16676	8.13
2-JW-A20	6817	4224	23708	12882	8.03
2-JW-A30	3693	2486	14472	8209	7.55
4-JW-A10	5164	2508	12401	6999	6.92
4-JW-A20	5654	2476	12144	7036	6.38
4-JW-A30	6564	2825	12037	6949	6.83
4-WD-B10	4328	2723	18076	8937	7.57
4-WD-B20	6528	4020	24534	12379	7.95
4-WD-B30	6497	4376	27260	14114	8.13
6-WD-A10	3638	1299	4522	3047	6.41
6-WD-A20	6087	2507	11581	6638	6.62
6-WD-A30	6275	2912	14904	8335	7.17
6-WD-B10	5478	2890	15339	7988	7.40
6-WD-B20	5568	2917	15110	8172	7.50
6-WD-B30	4953	2446	13454	6921	6.92
R2-JJK-A10	4981	3329	22830	11002	7.85
R2-JJK-A20	4074	2762	17486	9221	7.67
R2-JJK-A30	4615	3229	21301	11129	7.86
R4-YP-A10	4843	2682	16782	9393	7.38
R4-YP-A20	5842	3263	20048	10124	7.62
R4-YP-A30	4778	2684	15597	7425	7.45
R4-YP-B10	4229	2371	14848	7626	7.30
R4-YP-B20	5489	2918	16863	8495	7.42
R4-YP-B30	4260	2647	16588	8356	7.50
R6-YP-B10	5348	2444	11694	6477	7.05
R6-YP-B20	4981	2414	12380	6764	7.16
R6-YP-B30	5939	2857	14369	7868	7.29

**Fig 3:**
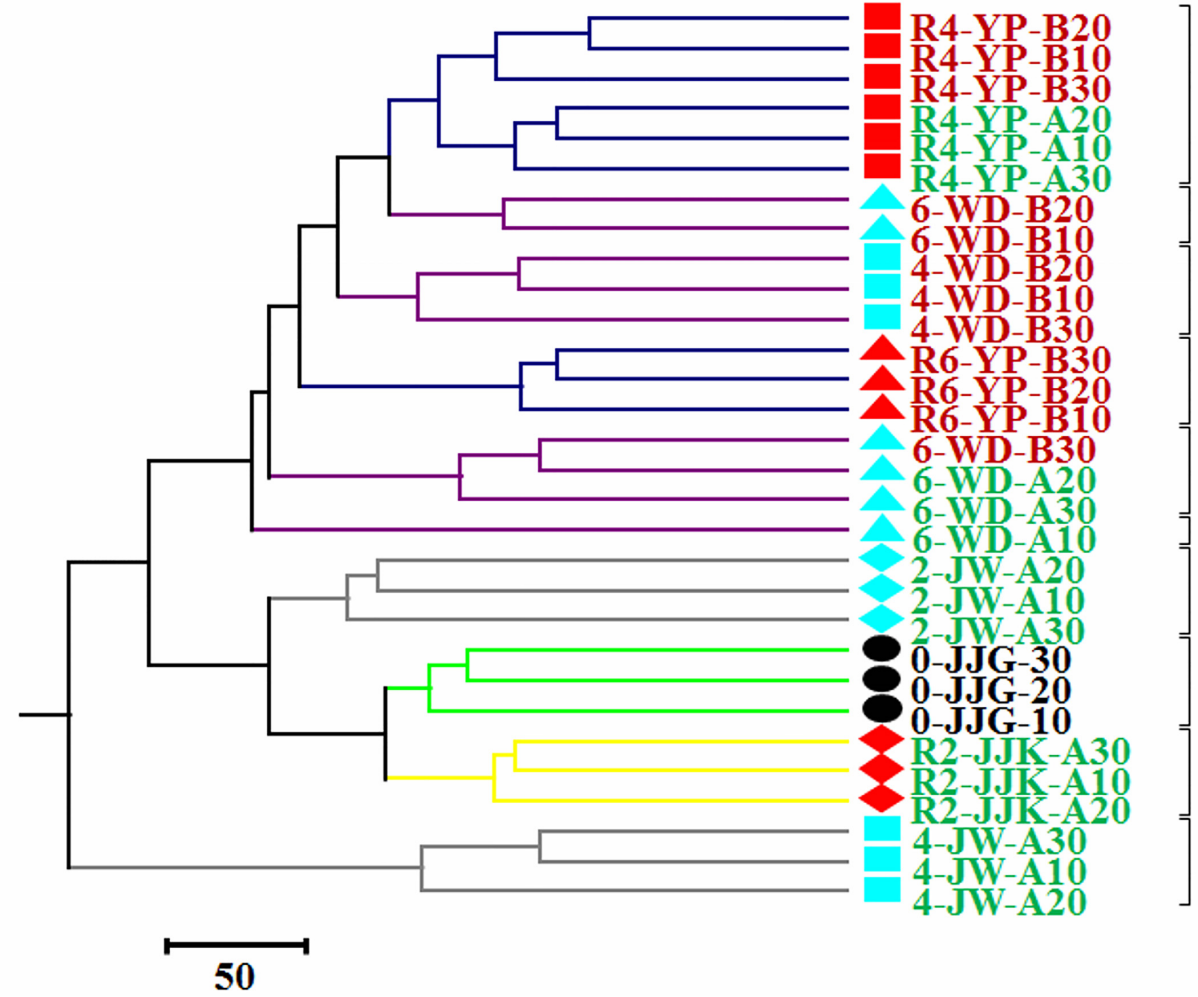
Unweighted-pair-group method with arithmetic-mean (UPGMA) tree. The UPGMA tress was generated from 1,000 jackknife iterations based on the non-normalized weighted UniFrac calculation. Dark blue, purple, grey, green, and yellow branches represent to soil samples from the Yulpori, Wondangri, Juwonri, Jajangri, and Jajakri areas, respectively. The circle, diamond, square, and triangle symbols indicate non-cultivated, 2-year-old, 4-year-old, and 6-year old soil samples, respectively. Black shapes represent non-cultivated samples, aqua is cultivated in the first round, and red is cultivated in the second round. Green font represents healthy samples and red represents unhealthy samples.

The time of cultivation may derive significantly bacterial diversity in 30 soil samples. An unweighted-pair group method using an arithmetic means (UPGMA) tree was generated from 1,000 jackknife iterations based on the non-normalized weighted UniFrac calculation (Fig 3). The 4-year-old soil samples from Juwonri (4-JW-A) were an out-group relative to the other samples, possibly due to the predominance of the phylum Chloroflexi. The level of branching was strongly categorized by years of ginseng cultivation, such as group I included the non-cultivated (Y0) and the 2-year-old (Y2) samples, and group II included 4-year-old (Y4) and 6-year-old (Y6) soil samples. Based on hierarchical clustering, no significant differences were observed between the bacterial communities present in soil samples obtained at different depths.

### Overall bacterial communities

**Fig 4:**
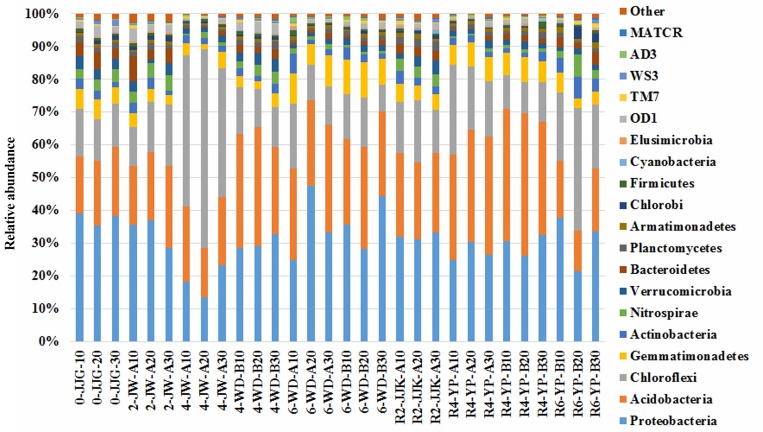
Relative abundance of bacterial phyla in 30 soil samples. Relative abundances are reported as percent of total bacterial sequences observed per samples. The other category includes phyla showing a percentage of reads <2% of the total reads in all of the soil samples.

Pyrosequencing data revealed great bacterial diversity in the 30 soil samples examined. The bacterial sequences were affiliated with 20 formally described phyla, 18 candidate phyla, and several unclassified lineages. Candidate phyla are those that lack cultivated members and are typically known only from 16S rRNA gene sequence data [24]. The most diverse Proteobacteria, Acidobacteria, and Chloroflexi phyla were detected in the 30 soil samples (average relative abundance 30.16%, 29.16%, and 18.15% of all samples, respectively), followed by the phyla Gemmatimonadetes (5.59%), Actinobacteria (2.28%), Verrucomicrobia (2.34%), Nitrospirae (2.09%), Bacteroidetes (2.07%), OD1 (1.52%), and Planctomycetes (1.40%) (Fig 4 and S3 Table). Other phyla, such as Chlorobi, Firmicutes, TM7, WS3, and AD3, were found at >1% in relative abundance in only one of the 30 soil samples. All remaining phyla were found at <1% in relative abundance in all of the samples. Chloroflexi was the dominant phylum in the 4-JW-A group (48.84%). Acidobacteria was the most abundant phylum in the R4-YP-A, R4-YP-B, and R6-YP-B groups, which were collected from the Yulpori area.

### Correlation of bacterial classes with soil chemical properties

**Fig 5:**
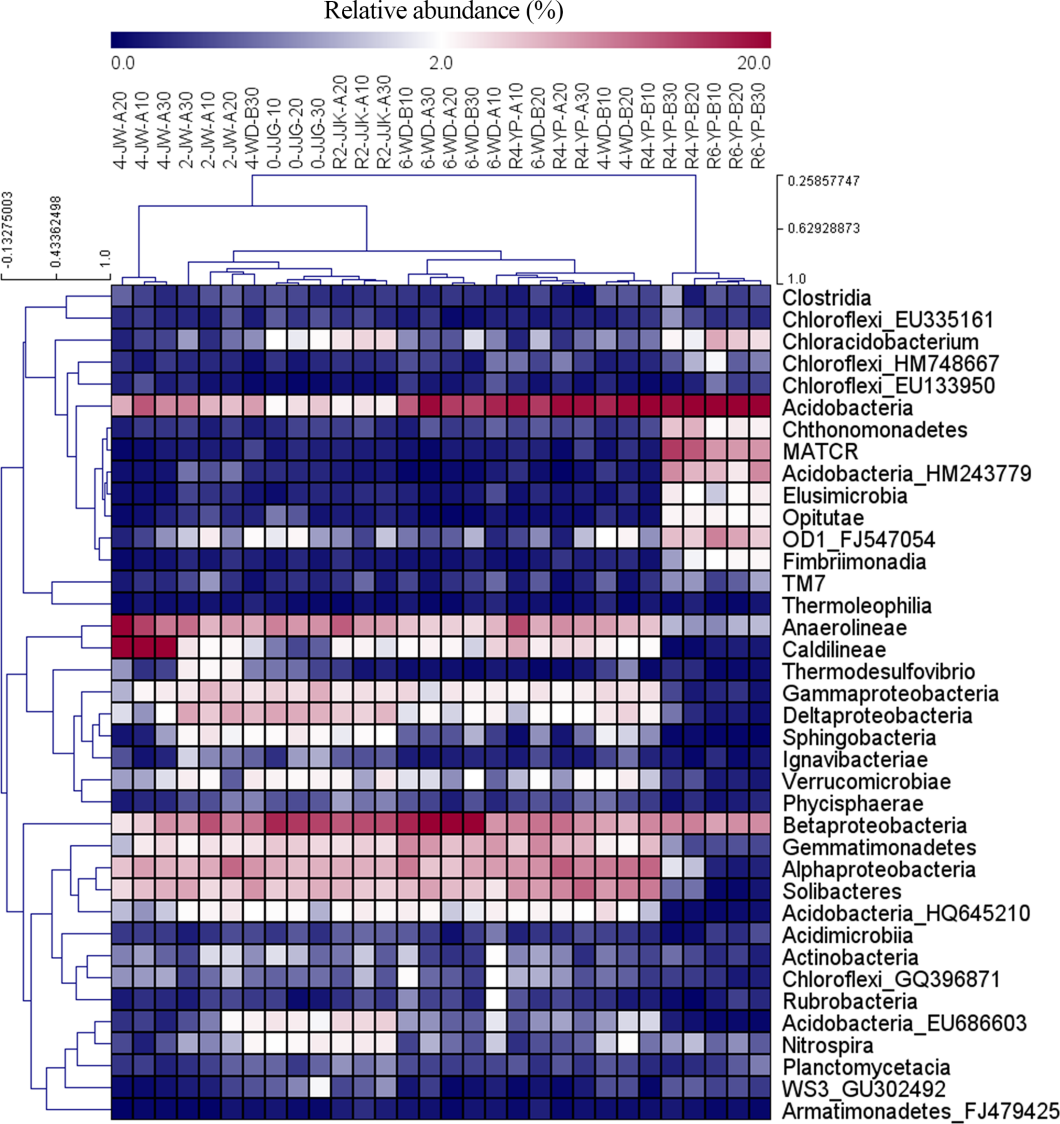
Heat map analysis showing the distributions of the core bacterial classes.

The “core microbiome”, as described by Shade & Handelsman [32], consists of microbial taxa present in all soil samples. This study found 38 classes that were present in all soil samples. These classes represented 90.07% to 97.60% of the relative abundance of all sequence reads (Fig 5 and S4 Table). The bacterial communities were dominated by Acidobacteria (average of 14.91%) followed by Betaproteobacteria (12.74%), Anaerolineae (9.23%), Alphaproteobacteria (9.47%), and Solibacteres (8.08%).

**Fig 6:**
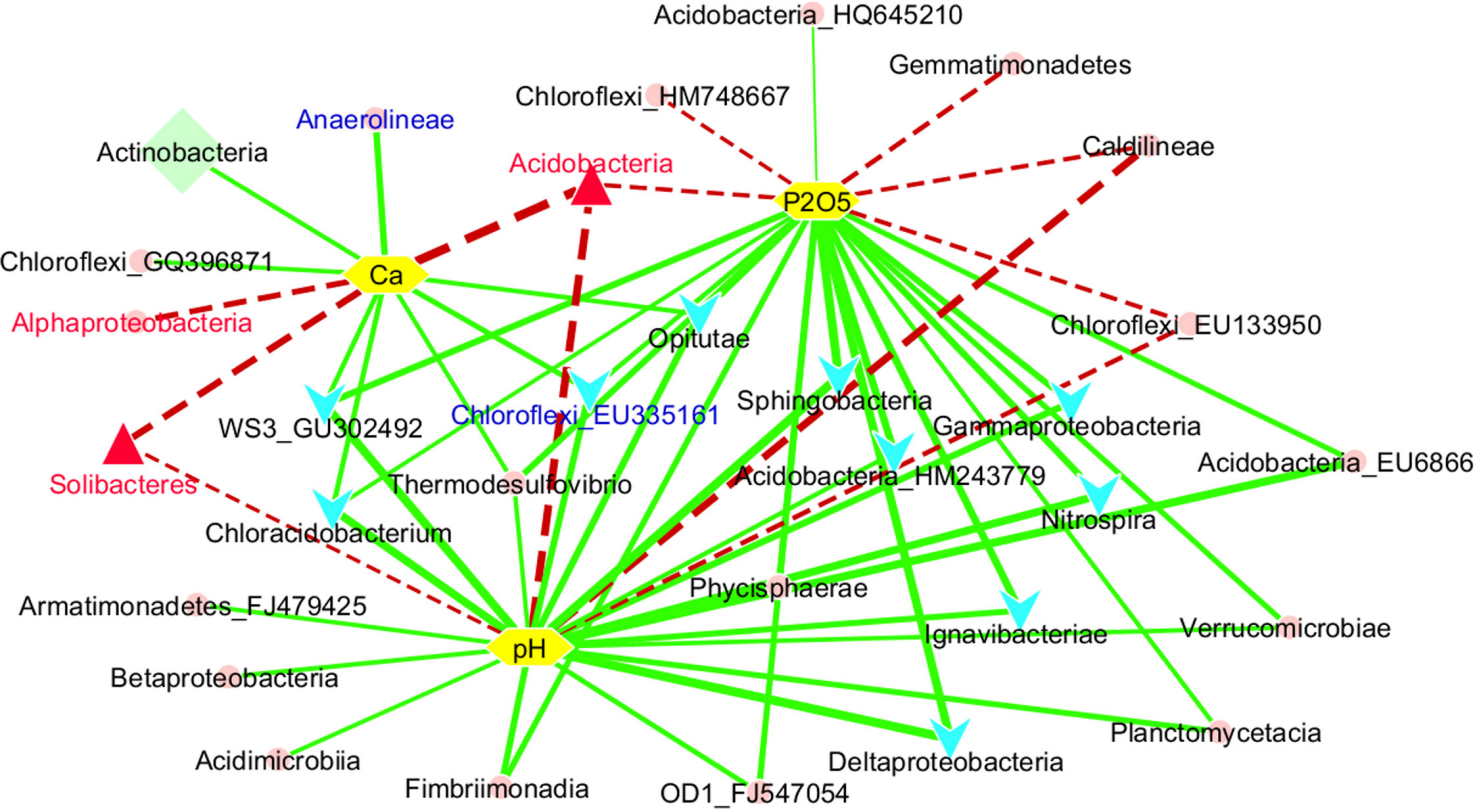
Network-inferred correlations of bacterial classes to soil parameters. Soil parameters (yellow hexagons) included soil pH, available phosphorus (P_2_O_5_), and exchangeable Ca^2+^. Aqua V-shapes indicate the classes that decreased with cultivation time, and the red triangles indicate the classes that increased with cultivation time. The light green diamond (Actinobacteria) indicates changes associated with soil depths. Red and blue fonts indicate the classes that were more and less populous, respectively, in unhealthy samples compared to healthy samples. The solid green lines indicate positive correlations while dashed red lines indicate negative correlations. Edge width is the correlation value. All correlations are significant at *p*<0.05 (one-way ANOVA, *post hoc* Tukey’s test).

To test and verify the effects of environmental variables on bacterial classes, correlation analyses of these factors and the relative abundance of 38 classes were performed using Spearman’s correlation (S5 Table). Soil pH strongly correlated with the prevalence of 24 of the 38 classes; in particular, pH correlated with Deltaproteobacteria, Sphingobacteria, Acidobacteria, and Caldilineae at r = 0.82, r = 0.81, r = -0.74, and r = -0.72, respectively. Available phosphorus (P_2_O_5_) correlated significantly with the prevalence of 23 of the 38 classes, with the highest correlations found with Sphingobacteria (r = 0.80) and Deltaproteobacteria (r = 0.77). Exchangeable Ca^2+^ exhibited a correlation with prevalence of 11 of the 38 classes, with the highest correlation to the class Acidobacteria (r = -0.86). Thus, we selected three factors (pH, available phosphorus (P_2_O_5_), and exchangeable Ca^2+^) to delineate the network of correlation of these factor to the bacterial classes, as shown in Fig 6.

Only Actinobacteria showed a significant difference between soil depths of 0–10 cm and 20–30 cm, with the highest observed population at the 0–10 cm soil depth.

### Core OTUs

Only three individual OTUs were found in all soil samples (S6 Table), accounting for 3.38% to 7.81% of the community reads in each sample. OTU-1 and OTU-2 belong to the family *Solibacteraceae* (phylum Acidobacteria), and the OTU-3 belongs to the genus *Pseudolabrys* (class Alphaproteobacteria). Interestingly, the numbers of these OTUs were similar between soil samples. Only OTU-2 demonstrated a significant difference between the healthy and unhealthy groups (*p* = 0.01, F = 7.2; PERMANOVA test).

### Core and distinct families

**Fig 7:**
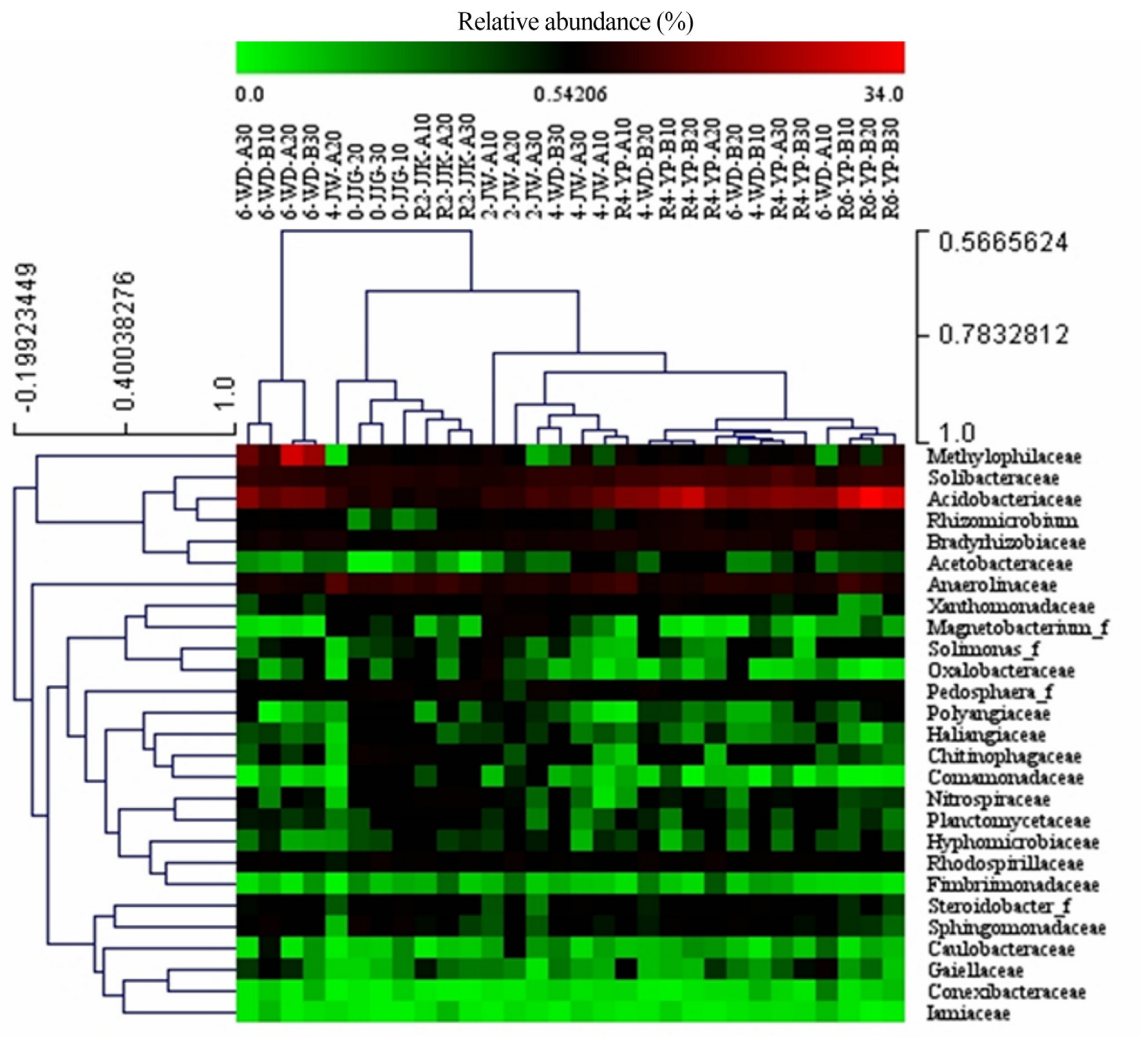
Heat map visualization of the distributions of core assigned families. The assigned families composing the core microbiota of the ginseng soil samples, with up to 46.9% relative abundance.

A total of 74 bacterial families were detected, including 27 assigned families and 47 uncultured families present, which contributed to 45.70% and 28.18%, respectively, of the relative abundance of all sequences (Fig 7 and S5 Table). These assigned families belong to the *Acidobacteria* (21.87%), *Proteobacteria* (15.65%), *Chloroflexi* (5.20%), *Verrucomicrobia* (1.29%), *Nitrospirae* (1.11%), *Bacteroidetes* (0.63%), *Actinobacteria* (0.51%), *Planctomycetes* (0.51%), and *Armatimonadetes* (0.14%) phyla. The top ten assigned families are *Acidobacteriaceae* (14.10%), *Solibacteraceae* (6.49%), *Anaerolinaceae* (5.35%), *Methylophilaceae* (3.93%), *Bradyrhizobiaceae* (3.13%), *Rhizomicrobium* (1.47%), *Pedosphaera* (1.34%), *Rhodospirillaceae* (1.24%), *Xanthomonadaceae* (0.96%), and *Steroidobacter* (0.85%).

Rare families were defined as those present only in one sample site. There were 34 rare families from 10 sample sites, including *Beutenbergiaceae*, *Alysiosphaera*, *Paludibacter*, *Demequinaceae*, *Thiobios*, *Luteolibacteria*, *Tsukamurellacea*, *Marivirga*, *Meganema*, *Syntrophomonadaceae*, *Microthrix*, *Bacteriovoraceae*, *Rhodobacteraceae*, and *Planktophila* (S8 Table).

### Core bacterial communities are affected by geographic area, years of cultivation, rounds of cultivation, and health states of the plant

**Fig 8:**
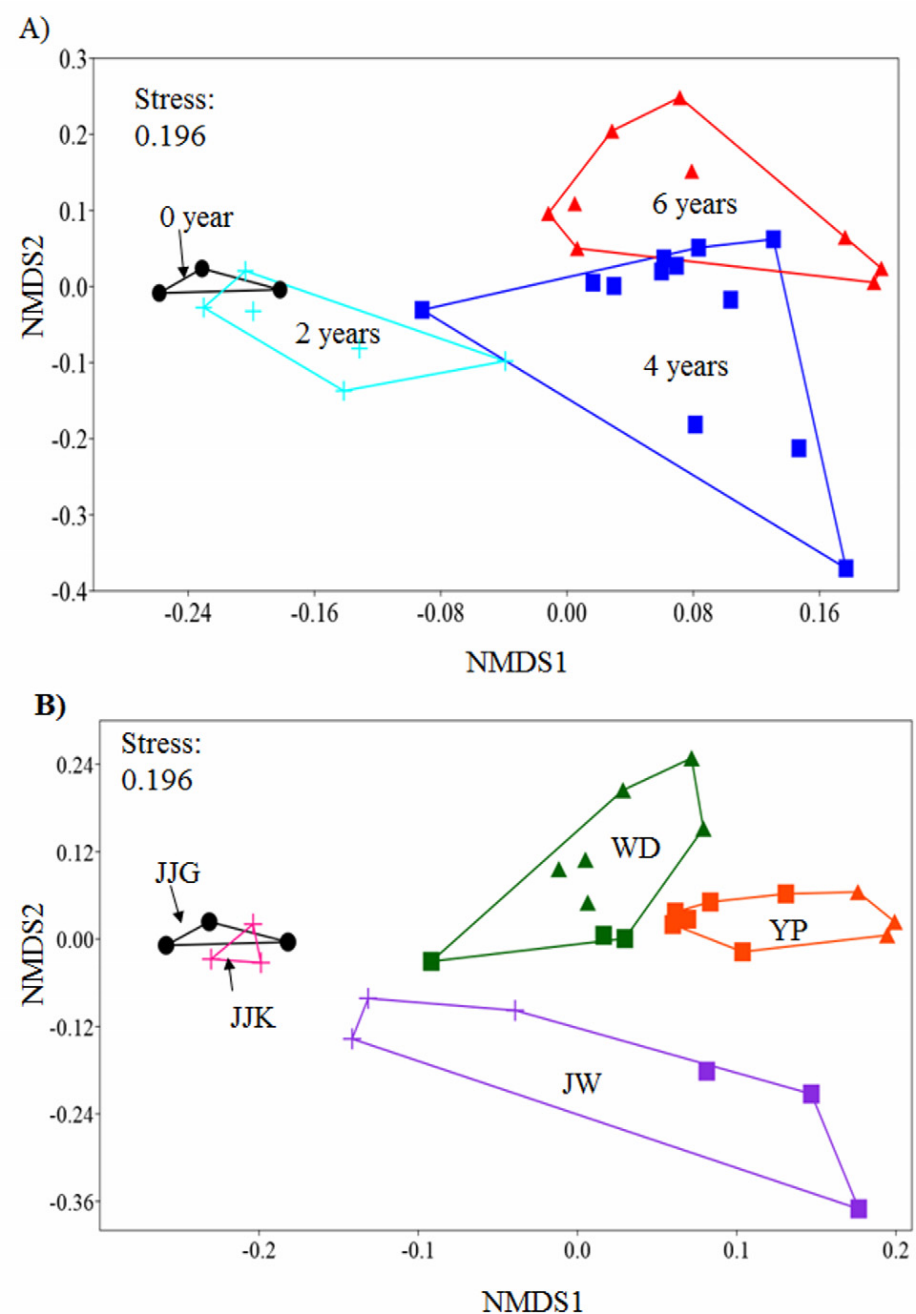
Non-metric multidimensional scaling (nMDS) plots of the core bacterial families in 30 soil samples. (A) nMDS plots show dissimilarities between cultivation years: non-cultivated (black), 2-year-old (aqua), 4-year-old (blue), and 6-year-old (red) samples. (B) nMDS plots show dissimilarities based on geography: Jajangri (JJG, black), Jajakri (JJK, pink), Wondangri (WD, darkgreen), Juwonri (JW, violet), and Yulpori (YP, orange).

Non-metric multidimensional bacterial composition scaling plots showed a clear separation in the ordination space based on time (Fig 8A) and geographic area (Fig 8B). We, therefore, suggested that geographic area and time of cultivation may be the main factors resulting in a shift in bacterial community structure. This was supported by statistical significance in PERMANOVA analyses (Table 2). The largest differences were found between the Jajangri and Yulpori soil samples (*p*<0.01, F = 15.68) and between the 2-year-old and 6-year-old soil samples (*p*<0.001, F = 8.397). PERMANOVA analyses also revealed that the bacterial communities may be significantly distinct between rounds of cultivation and based on the health states of the ginseng. No significant difference associated with soil depth was detected.

**Table 2:** PERMANOVA analysis of the effect of geographic area, years of cultivation, rounds of cultivation, depths, and health states of plant samples.

Variations	Target	*p*-value	F-value
Geography (Jajangri, Juwonri, Wondangri, Jajakri, and Yulpori)	Jajangri-Juwonri	0.02538*	4.04
	Jajangri-Wondnagri	0.0061**	6.84
	Jajangri-Jajakri	0.0999	2.97
	Jajangri-Yulpori	0.0045**	15.68
	Juwonri-Wondangri	0.0002***	5.17
	Juwonri-Jajakri	0.024*	3.96
	Juwonri-Yulpori	0.0001***	7.72
	Wondangri-Jajakri	0.0044**	6.94
	Wondangri-Yulpori	0.0002***	4.45
	Jajakri-Yulpori	0.0041**	15.70
Time (year 0, 2, 4, or 6 of ginseng cultivation)	0–2	0.048*	1.92
	0–4	0.0047**	6.04
	0–6	0.0046**	8.03
	2–4	0.0003***	5.95
	2–6	0.0003***	8.40
	4–6	0.0166*	2.80
Rounds of cultivation (Non-cultivated, first, second round)	Non-cultivated-first	0.0269*	2.73
	Non-cultivated-second	0.0051**	15.68
	First-second	0.0002***	4.83
Depths (0–10, 10–20, 20–30 cm)	0–10 cm vs.10-20 cm	0.9955	0.16
	0–10 cm vs. 20–30 cm	0.7244	0.64
	10–20 cm vs. 20–30 cm	0.9774	0.28
Health states of ginseng (healthy and unhealthy)	Healthy-Unhealthy	0.0048**	3.19

PERMANOVA tests were conducted using a Bray-Curtis similarity matrix of relative abundance of core bacterial composition with 9999 permutations.

*p≤0.05

**p≤0.01

***p≤0.001

SIMPER analyses of the core bacterial communities were calculated based on the overall percent contribution of each taxon to the average dissimilarity between groups. The list of taxa is shown in decreasing order with respect to its importance in discriminating samples (S9 Table). The SIMPER results showed that the average dissimilarity between different geographic groups was 37.26%. The first and the second rounds of cultivation had an average dissimilarity of 30.05%. The average dissimilarities between healthy and unhealthy soil samples, and year of cultivation were 35.01% and 37.02%, respectively. The highest level of dissimilarity was attributed to the family *Acidobacteriaceae*, which contributed approximately 20% of the dissimilarity between samples.

### Bacterial groups showed changes through cultivation time

To obtain more insight into changes in the bacterial populations over cultivation time, we compared the relative abundances of the core classes and families. The bacterial groups comprising of at least 1% of the total sequences in at least one sample, on average, were examined.

**Fig 9:**
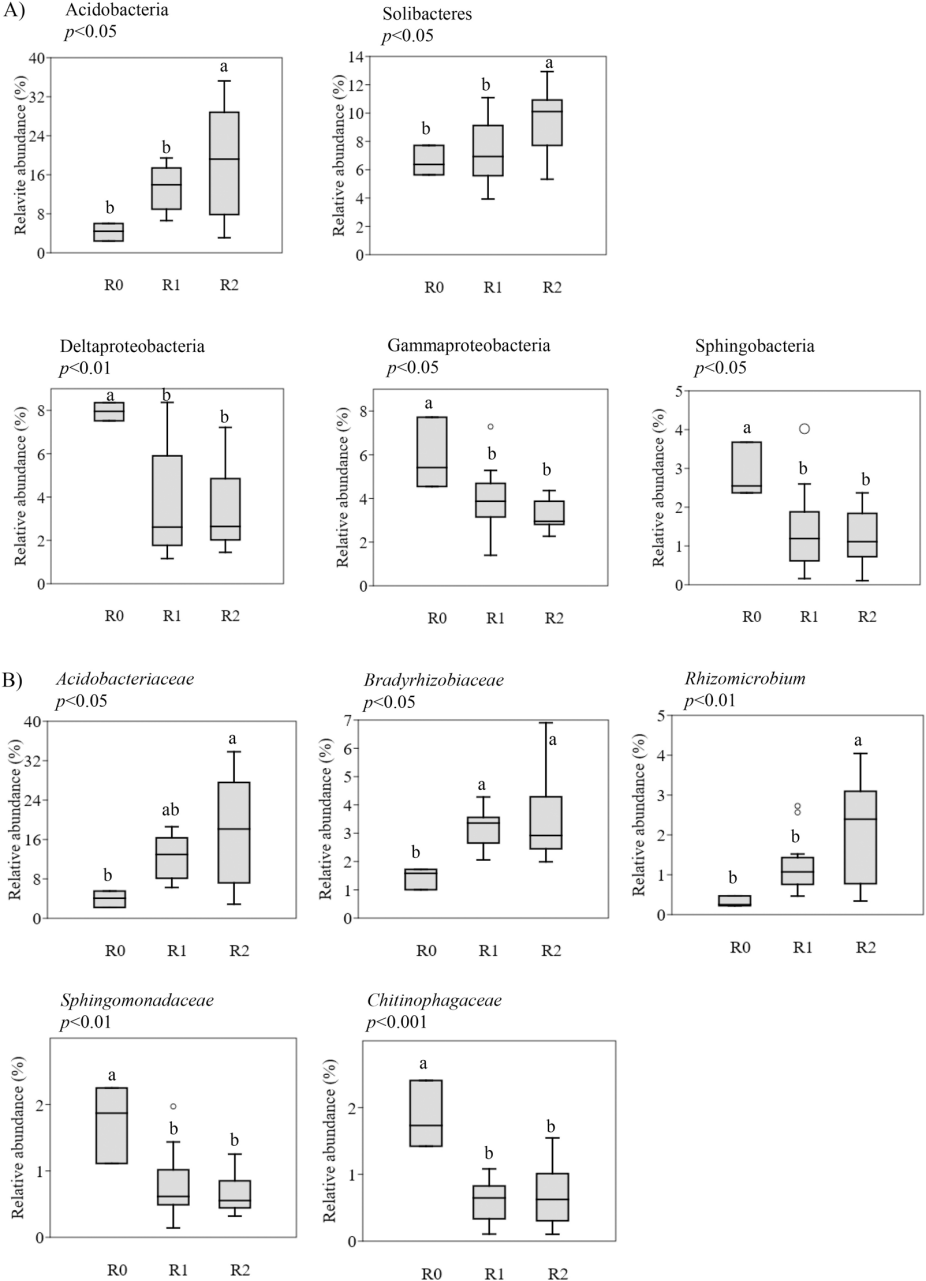
Changes in bacterial populations according to rounds of cultivation. **(**A) Boxplots indicate bacterial classes, (B) Boxplots indicate bacterial families. R0, non-cultivation; R1, first round of cultivation; R2, second round of cultivation. The *p*-values were generated by one-way ANOVA, *post hoc* Tukey’s test. “a” and “b” indicate significant differences between the round of cultivation (*p*<0.05).

First, shifts in the bacterial populations based on the round of cultivation were considered, including non-cultivated soil (R0), the first round soil (R1) and the second round soil (R2). At the class level (Fig 9A), Acidobacteria was found to have a significantly high relative abundance in R2 (average of 19,55%) and R1 (13.32%) samples compared to R0 (4.28%) samples (*p*<0.05, Tukey’s test). Solibacteres was found to be slightly more abundant in R2 (10.17%) samples than in R1 (8.96%) and R0 (7.54%) samples (*p*<0.05, Tukey’s test). Meanwhile Deltaproteobacteria, Gammaproteobacteria, and Sphingobacteria were determined to be more abundant in R0 (7.94%, 5.89%, and 2.86%, respectively) samples than in R1 (3.85%, 3.88%, and 1.41%, respectively) and R2 (3.31%, 3.25%, and 1.16%, respectively) samples (*p*<0.05, Tukey’s test). At the family level (Fig 9B), *Acidobacteriaceae*, *Bradyrhizobiaceae*, and *Rhizomicrobium* levels increased after ginseng cultivation, especially during the second round of cultivation. The levels of *Sphingomonadaceae* and *Chitinophagaceae* decreased after R1 and R2 ginseng cultivation compared to the R0 non-cultivated soil samples R0 (*p*<0.01, Tukey’s test).

**Fig 10:**
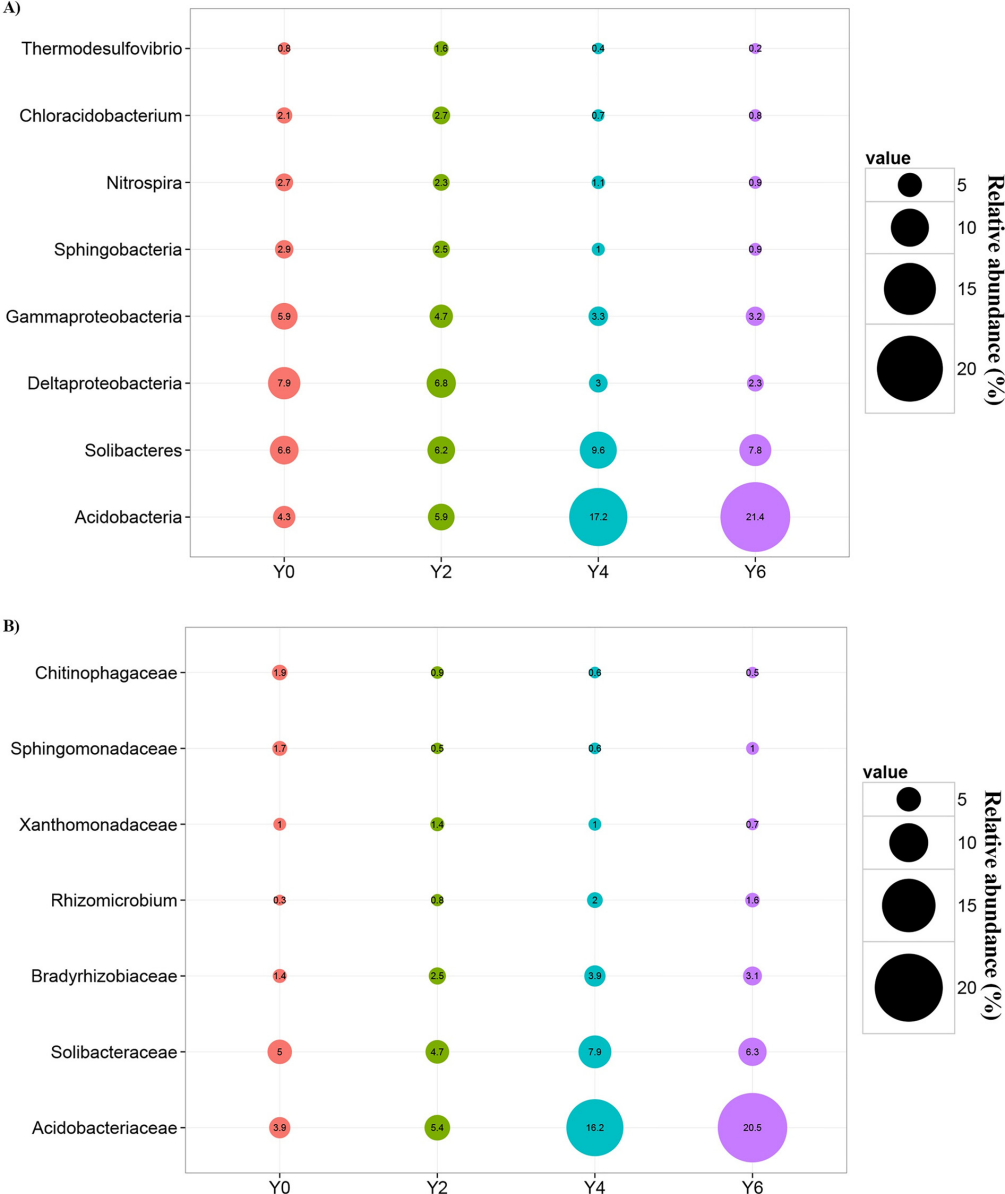
Changes in bacterial populations over years of cultivation. Bubbles represent the average relative abundances of bacterial populations at the class level (A) and the family level (B). 2-year-old (Y2, n = 6), 4-year-old (Y4, n = 12), 6-year-old (Y6, n = 9), and non-cultivated (Y0, n = 3) soil samples. All bacterial populations showed significant differences between cultivation times with *p*<0.05 (one-way ANOVA, *post hoc* Tukey’s test).

The ginseng cultivation times of soil could be divided as 2-year-old (Y2), 4-year-old (Y4), 6-year-old (Y6) and control non-cultivated (Y0). At the class level (Fig 10A), Acidobacteria increased significantly with increased cultivation time; however, Deltaproteobacteria, Gammaproteobacteria, Sphingobacteria, and Nitrospira decreased over time. The highest relative abundance of Solibacteres occurred in Y4 samples. On the other hand, Chloracidobacterium and Thermodesulfovibrio demonstrated higher relative abundance in Y2 samples compared to Y4 and Y6 samples. At the family level (Fig 10B), *Acidobacteriaceae* increased significantly over time. In constrast, *Chitinophagaceae* showed a successional decrease. *Solibacteraceae*, *Bradyrhizobiaceae* and *Rhizomicrobium* were most abundant in Y4 samples. The relative abundances of *Sphingomonadaceae* and *Xanthomonadaceae* showed were highest in Y0 and Y2 samples, respectively.

### Bacterial taxa in soil related to health states of ginseng

**Fig 11:**
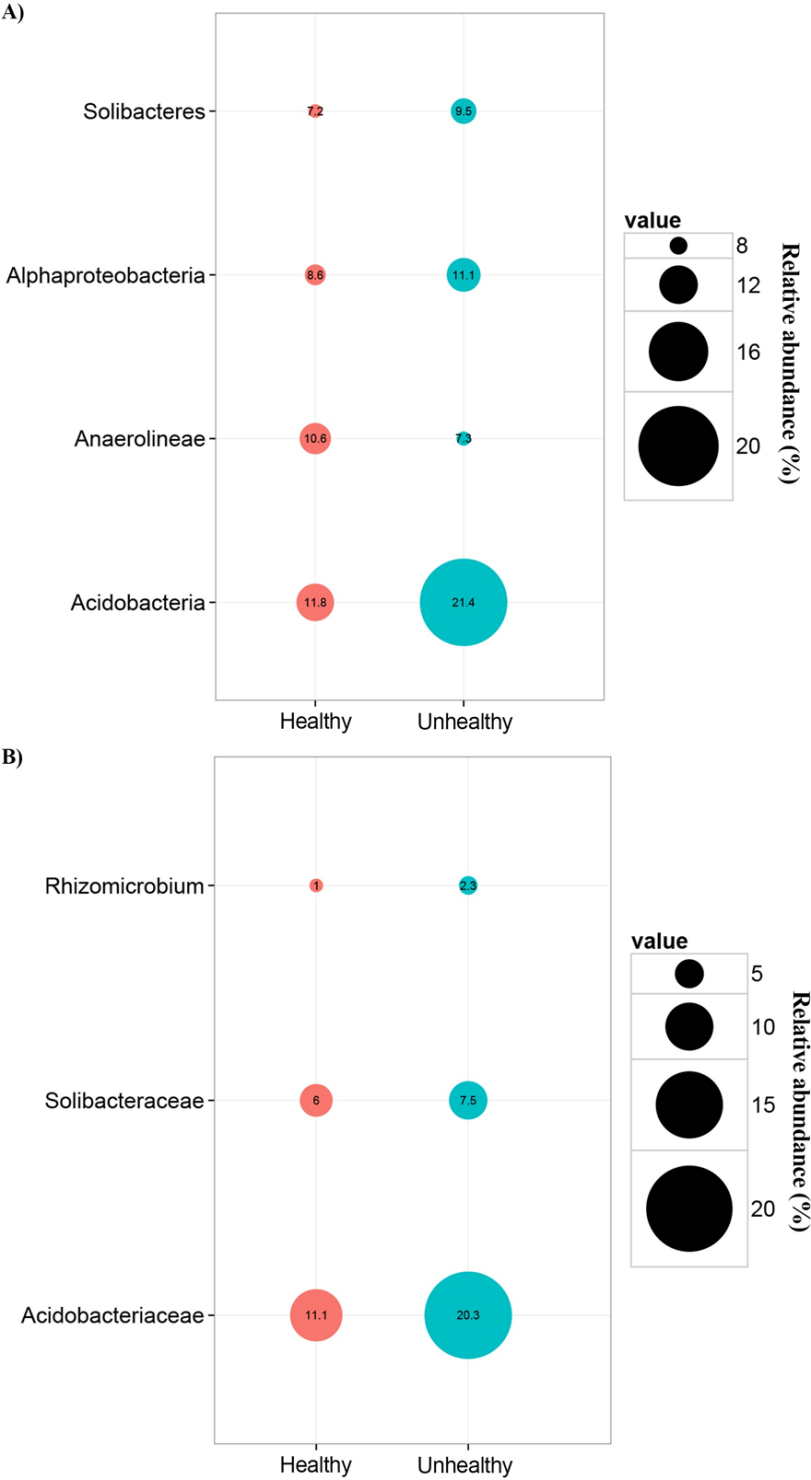
Healthy and unhealthy ginseng soil samples had significant differences in bacterial population. **(**A) At the class level, (B) At the family level.

To evaluate comparative differences between healthy and unhealthy ginseng soil samples, we utilized bacterial groups comprising at least 1%, on average, of the total sequence in at least one sample. At the class level (Fig 11A), Acidobacteria, Solibacteres and Alphaproteobacteria seemed significantly higher relative abundance in unhealthy soil samples than in healthy soil samples (*p*<0.05, Tukey’s test); Anaerolineae attained significant higher relative abundances in healthy soil samples than in unhealthy soil samples (*p* = 0.012, Tukey’s test). At the family level (Fig 11B), *Acidobacteriaceae*, *Solibacteraceae*, and *Rhizomicrobium* had significantly higher relative abundance in unhealthy soil samples, compared to healthy soil samples.

The bacterial species associated with healthy ginseng soil samples that were not detected in unhealthy ginseng soil samples may be considered to be beneficial bacteria. In contrast, species present in unhealthy ginseng soil samples that were not present in healthy ginseng soil samples possibly considered to be detrimental bacteria. Of the 341 total assigned species detected in this study, 244 species were found only in healthy ginseng soil samples, and 97 species were found only in unhealthy ginseng soil samples (S10 Table).

### Pathogenic Potential Bacteria Detected By Pyrosequencing

Based on the previously published list of phytopathogenic-related bacteria in plants [33–35], we could identify the family *Ralstonia*, in addition to the genera *Clostridium*, *Arthrobacter*, *Bacillus*, *Rhizobacter*, *Herbaspirillum*, *Streptomyces*, *Pseudomonas*, *Burkholderia*, (*Janthinobacteium*, *Nocardia*, *Rhodococcus*, *Acidovorax*, *Leifsonia*, and *Corynebacterium* in our data (S11 Table). In general, pathogenic potential bacteria were present at extremely low prevalence (0.5%-2.5%). Moreover, some genera only existed in one soil sample, such as *Acidovorax* (in 2-JW-A20), *Leifsonia* (in R2-JJK-A10), and *Corynebacterium* (in R2-JJK-A20).

## Discussion

The primary purpose of this study was to construct a comprehensive catalog of bacterial diversity and community structure in Korean ginseng cultivated soil. Soil is known to be one of the most diverse environments [36]. In addition, plants present in agricultural soil provide carbon, root exudates, and plant secondary metabolites [37] which require an enormous reservoir of soil bacteria. As expected, we obtained excellent data from 454 pyrosequencing (Table 1). Indeed, 454 pyrosequencing has been shown to be an efficient method to quantify the extent of microbial diversity in agricultural soil [15,38–39].

Our diversity analysis revealed a decreasing trend from 2-year-old to 6-year-old samples which supported the hypothesis that bacterial diversity would decrease over longer cultivation periods. This can be explained by the finding that older roots excreted low amounts of organic matter [11.40], or perhaps that soil nutrients such as carbon and nitrogen, were depleted by long-term cultivation. Therefore, long-term ginseng monoculture may cause nutrient-deficiency stress in the bacterial populations, which ultimately culminated in a decrease in diversity. On the basis of this study, we suggest that additional factors, such as phosphorus deficiency and lower pH in 6-year-old samples, may lead to less bacterial diversity compared to that in 2 and 4-year-old samples. Lower diversity after long-term ginseng monoculture suggests that only a few populations are able to adapt to changes in the soil environment. These findings are consistent with previous observations [11,40] which reported similarly reduced diversity in ginseng cultivated soil samples over time. The same pattern was observed in soil samples, evaluated using a high-throughput sequencing method, over a 7-year gradient of potato monoculture [41]. However, the numbers of OTUs and valid reads in this study were significantly greater than a previous study [40], which included 200 clones with cumulative counts of fewer than 50 OTUs. In addition, the Shannon index (6.38–8.13) in this study was significantly higher than the Shannon index in non-rhizosphere ginseng soil samples (0.21–0.57) [11] and in rhizosphere soil samples (0.54–0.67) [40]. The number of observed bacterial OTUs in ginseng soil (1,299–5,453 OTUs) was higher than that observed in a previous study by Eilers et al. (320–520 OTUs) [18] but lower than that in a study of German grass soil samples (4,78–6,231 OTUs) [16]. Consequently, the results presented here are in accordance with other studies [14–15,42].

The rarefaction curves in our study did not reach asymptotes. This indicates that the maximum detection limit of bacterial diversity was not achieved. In this study, the pyrosequencing of the 30 soil samples was conducted in parallel, therefore reducing the number of sequences read per sample and perhaps limiting the number of detectable OTUs. This pattern is in contrast to the earlier study of Li et al. [40], which demonstrated rarefaction curves near saturation (using an amplified ribosomal DNA restriction analysis method).

We detected 38 phyla in this study, which is higher than the number of phyla found in previous studies on rhizospheres as well as in bulk ginseng cultivated soil samples. The dominances of the phyla Proteobacteria and Acidobacteria were predicted since these groups have been detected as the majority phyla in other soil environments, such as forest soil [43–44], agricultural soil [39,45], and in ginseng soil [40]. As suggested by Smit et al. [46], the ratio between Proteobacteria and Acidobacteria may be indicative of the nutrient status in the soil ecosystem. In this study, the ratio between Proteobacteria and Acidobacteria in ginseng cultivated soil samples (approximately <1.6) was lower than in non-cultivated soil (approximately 1.9).This ratio was even lower in 4-year-old samples and 6-year-old samples during the second round of cultivation (0.8 and 0.5, respectively). These data may indicate that more oligotrophic conditions in the second round compared to the first round and to non-cultivated soils. In general, these results were consistent with the notion of Smit et al. [46] regarding the correlation between the ratio of Proteobacteria to Acidobacteria and nutrient status in soil. In this study, long-term ginseng cultivation may decrease a number of nutrients in the soil. The acidobacterial groups can utilize a broad range of substrates [47] and tend to prefer oligotrophic niches [48]. Therefore, the phylum Acidobacteria, including the classes Acidobacteria and Solibacteres, showed a particularly evident increase over time during ginseng cultivation. However, some acidobacterial groups have been reported to prefer copiotrophic condition in the grassland and forest soils [49], and the snow-accumulating soils [50]. Therefore the increasing of Acidobacteria can be explained based on their acidic tolerance or preference. In this study ginseng soil undergoes slight acidification over time (S2 Table). The more acidic is the environment, the more abundant are the Acidobacteria, since they prefer living in such conditions. This raises the question of which nutrient deficiency is related to the relative abundance of the acidobacterial groups. Available phosphorus and exchangeable Ca^2+^ showed significant negative correlations with class Acidobacteria (S5 Table and Fig 6). In contrast to Acidobacteria, Deltaproteobacteria and Gammaproteobacteria, which dominated phylum Proteobacteria, decreased over time with ginseng cultivation. The decreased abundance of Gammaproteobacteria in ginseng soil is assumed to be due to its preferred lifestyle, because this bacterial group is considered a copiotrophic bacterium that grows slowly in low nutrient conditions [51].

Interestingly, the abundant presence of the phylum Chloroflexi in this study was not reported in previous studies [13,40] that employed traditional methods to investigate bacterial communities in ginseng cultivated soil samples. Members of this phylum were found to be the major bacterial population in paddy soil [45,52]. The soil samples in this study were originally paddy soil samples that were later used to cultivate ginseng. Therefore, a decipherable prevision of the presence of Chloroflexi was expected in our samples. However, the abundance of this phylum in the 4-JW-A and R6-YP-B groups was unexpected. The major classes of *Caldilineae* and *Anaerolineae* within the *Chloroflexi* phylum are known to mediate anaerobic ammonium oxidation (anammox). Kindaichi et al. [53] reported the coexistence of uncultured *Caldilineae* and *Anaerolineae* in an anammox reactor fed with synthetic nutrient medium (without organic carbon compounds) over a 2-year period. These classes also can degrade and utilize cellular compounds derived from dead biomass and metabolites of anaerobic ammonium oxidation bacteria. Since ginseng soil is a poor source of nutrients, *Caldilineae* and *Anaerolineae* may be useful bacteria in that they can increase the levels of nutrients in the soil. Moreover, the prevalence of *Anaerolineae* has been shown to be significantly increased upon co-culture with methanogens [54]; since the 30 soil samples we analyzed contained a large amount of archaeal methanogens (data not shown), the prevalence of *Anaerolineae* in ginseng soil was high. We also believe that the abundance of Chloroflexi could be related to other factors, such as syntrophic oxidation of butyrate [55] and presence of hydrogen-consuming methanogens [56].

Other bacterial phyla, including Gemmatimonadetes, Planctomycetacia, Actinobacteria, Verrucomicrobia, Firmicutes, and Bacteroidetes which were found in previous studies [13,40] were also present with relative abundance >1% in our data. The other newly identified cultured representative phyla in ginseng soil include Nitrospirae, Armatimonadetes, Chlorobi, Cyanobacteria, Elusimicrobia, Spirochaetes, Fibrobacteres, Lentisphaerae, Tenericutes, Aquificae, and Caldiserica, as well as 18 additional candidate phyla.

Some previous studies have detected core OTUs in bacterial soil communities [57–59]. The core OTUs in this study belonging to Solibacteraceae and Alphaproteobacteria were in agreement with previous studies. However, at the lower level, the family *Solibacteraceae* and the genus *Pseudolabrys* seemed to be distinct for ginseng soil samples. The families *Acidobacteriaceae*, *Sphingomonadaceae*, *Anaerolinaceae*, *Rhodospirillaceae*, *Nitrospiraceae*, *Chitinophagaceae*, and *Planctomycetaceae* were identified as part of the core microbiome in samples from pristine forest and 8-year-old grasslands surrounded by the same forest [57], *Bradyrhizobiaceae* and *Xanthomonadaceae* have been identified as part of the core phyllosphere microbiome in neotropical forests [59]. Therefore, it is not surprising that these particular families appeared in ginseng cultivated soil. Interestingly, *Sphingomonadaceae* belonging to the order Sphingomonadales was also found in 1- to 4-year-old ginseng rhizospheres [40]. Therefore, *Sphingomonadaceae* is likely a resident taxon in ginseng soil samples and is easy to isolate either by culture- or nonculture-dependent methods.

We expected that soil depth from 0–30 cm would influence the bacterial communities. However, only Actinobacteria was observed to have significant differences between the 0–10 cm and 20–30 cm soil depth samples. Actinobacteria are known as decomposers of dead plant biomass [60–62]. Therefore, members of the actinobacterial group likely prefer the upper subsoil (0–10 cm), which contains more dead plant biomass compared to the lower subsoil (20–30 cm).

To our knowledge, this is the first study that identified significant differences in bacterial community structure between geographic areas, cultivation rounds in ginseng fields, and the health state of the ginseng. These results are generally consistent with many previous studies that have examined bacterial communities in soil based on geographic area [63–66]. Some studies have focused on the effect of time on soil microbial communities, especially with monoculture methods. Habekost et al. [67] discovered a few significant effects four years after the establishment of their experiment, and Liu et al. [41] found significant correlations between bacterial taxa and the year of monoculture in potato soil samples. Soil microbial communities were affected by the continuous cropping of rice [68], peas [69], watermelon [68], and peanut [70]. Our study provides additional support for the hypothesis of Chen et al. [71], who suggested that “successional changes in soil microbial communities with continuous cropping could be a common feature.” Healthy and unhealthy ginseng soil samples across residences harbor different bacterial communities. This result supports our knowledge of bacterial populations related to good and poor soil, as described by Figuerola et al. [72]. The composition of bacterial communities was similar between soil samples. Therefore, we only considered the effect of these factors based on changes in relative abundance. Our explanations for the different relative abundances of Acidobacteria (dominated by the family *Acidobacteriaceae*), Deltaproteobacteria, and Gammaproteobacteria (included *Xanthomonadaceae*) over cultivation time are described above. The observed decrease in Sphingobacteria (included *Chitinophagaceae*) over cultivation time can be explained by their copiotrophic lifestyle [48]. Interestingly, we observed the different trend for bacterial groups of the class Alphaproteobacteria. The family *Sphingomonadaceae* was found to have decreased in number after ginseng cultivation compared to non-cultivated soil; however, both *Bradyrhizobiaceae* and *Rhizomicrobium* increased in abundance. This finding can be explained as the ginseng root exudate favored nitrogen-fixing bacterial groups (*Bradyrhizobiaceae* and *Rhizomicrobium*) but not those groups that utilize aromatic compound degradation (*Sphingomonadaceae*). The soil chemical compositions found in our results were in agreement with the range of soil chemical properties for ginseng cultivation reported by Yeon et al. [73]. The correlation network demonstrated that many of the abundant classes strongly responded to pH, available phosphorus, and exchangeable Ca^2+^ (Fig 6). Soil pH was shown to be the strongest driver shaping bacterial community structure, and these results correspond to previous studies of soil samples [17, 42,44,48,74–78]. Available phosphorus has been shown to have effects on microbial community structure [42,79]. Long-term cultivation of ginseng is known to generate acidic soil, which can cause phosphorus absorption by Fe-Al and Ca^2+^ [80–82], leading to low soil concentrations of phosphorus (<100 mg P_2_O_5_ kg^-1^, S2 Table). The amount of available phosphorus limits microbial activity [83], biomass [84–86], and microbial growth [85,87]. Therefore, the amount of available phosphorus in soil is likely one of the crucial factors in shaping microbial communities in ginseng soil and that phosphorus should be adequately supplied in the cultivation of ginseng. Exchangeable Ca^2+^ was one of the most important factors affecting bacterial composition. This result is supported by Singh et al. [88], where exchangeable Ca^2+^ showed a greater effect on bacterial population than did pH. Another study by Sridevi et al. [79] showed that the bacterial community structure in calcium-supplemented soil samples was significantly different from that of reference soil samples.

Using this high throughput sequencing method, the individual taxa present in soil that could not be observed by previous methods were investigated. Based on the list of target healthy species and unhealthy species, researchers can potentially design a management approach to control the presence of bacterial species in the soil and improve ginseng productivity. In addition, many species are still not classified as either pathogenic or non-pathogenic. Therefore, information on the bacterial sources in healthy and unhealthy soil samples is important for overcoming this problem. However, additional research is needed to evaluate the pathogenic or beneficial properties of these species to ginseng.

Among the phytopathogenic potential bacteria identified in this study, the genera *Sphingomonas*, *Arthrobacter*, *Bacillus*, and *Pseudomonas* have been frequently isolated under laboratory conditions in the previous studies [89–92]. Many species belonging to the genus *Sphingomonas* have been shown to be able to convert ginsenosides [89–90]. Consequently, ginseng soil appears to be an ideal habitat for *Sphingomonas*. In addition to causing disease, bacteria in the genus *Sphingomonas* can also cause microbiological corrosion, produce exopolysaccharide polymers, and biodegrade refractory organic compounds [91]. Therefore, further study should be conducted to determine the correlation of *Sphingomonas* spp. with the health state of ginseng. The genus *Clostridium* was detected in almost all of our soil samples; however, members of this genus are anaerobic and could therefore not be detected under our aerobic isolation conditions. The genera *Acidovorax*, *Leifsonia*, and *Corynebacterium* were rarely observed in the 30 soil samples, a finding that is in agreement with the isolation data [92]. Of the top 10 plant pathogenic bacteria [93] and the common bacterial diseases known to affect ginseng [94–95], only the genus *Pseudomonas* and the family *Ralstonia* were found in our samples. It is speculated that additional pathogenic bacteria do exist in ginseng soil, but that these bacteria could not be isolated by our culture-dependent methods.

## Conclusion

This study highlighted the bacterial diversity and community structure in ginseng cultivated soil samples. Bacterial diversity richness decreased over the years of cultivation. Bacterial compositions fluctuated depending on cultivation time and the health state of the ginseng. These bacterial reservoirs responded to changes in the soil conditions after long-term monoculture of ginseng (e.g., nutrient deficiency, low pH, changes in exudates). This process occurred more rapidly in the second round of cultivation. Based on the presence of different bacterial populations between healthy and unhealthy ginseng soil samples, as well as with different cultivation time, we were able to determine the specific bacterial taxa related to the health state of soil samples. However, additional studies will be needed to elucidate how these bacteria affect ginseng roots. The ability to detect beneficial and detrimental bacterial populations could be a promising advance for the development of ginseng soil management programs for the improvement of sustainable ginseng production.

## Supporting Information

S1 Fig Pairplot of chemical explanatory variables. The lower diagonal panels contain the (absolute) correlation coefficients, and the upper diagonal panels contain scatterplots (and smoothing line was added). The font size of the cross-correlation is proportional to the value. Black, green and red circles indicated non-cultivation, first round and second round soil samples, respectively.
(TIF)

S1 Table Description of the 30 soil samples. Soil samples were collected in the city of Paju city and in Yeoncheon County (Gyeonggi-do, South Korea).
(DOCX)

S2 Table Edaphic properties of all soil samples. EC, electrical conductivity; OM, organic matter.
(DOCX)

S3 Table Relative abundance of bacterial phyla in 30 soil samples. Relative abundances are reported as the percent of total bacterial sequences observed per samples.
(XLSX)

S4 Table Bacterial classes present in all soil samples. Relative abundances are reported as the percent of total bacterial sequences observed per samples.
(XLSX)

S5 Table Spearman’s rank correlations between the relative abundances of bacterial classes and the soil chemical compositions in all soil samples. EC, electrical conductivity; OM, organic matter. Bold number: *p*<0.05; Bold and single underline numbers *p*<0.01; Bold and double underline numbers *p*<0.001.
(DOCX)

S6 Table Core OTUs occurring in all soil samples. The total number of reads of OTUs and taxonomic assignment are reported for each OTU.
(DOCX)

S7 Table Shared assigned bacterial families present in all soil samples. Relative abundances are reported as the percent of total bacterial sequences observed per samples.
(XLSX)

S8 Table Rare assigned families at each site. 0, 2, 4, 6, R2, R4 and R6 indicate 0, 2, 4, and 6 years at first and second cultivation, respectively. JJG, Jajangri; JW, Juwolri; WD, Wondangri; JJK, Jajakri; YP, Yulpori; A, healthy soil; B, unhealthy soil.
(DOCX)

S9 Table SIMPER analyses. Results of SIMPER analyses indicating the contribution of the core families to observed differences in community structure among geographic area (S7A Table), cultivation round (S7B Table), time (S7C Table), and health states of the plant (S7D Table).
(XLSX)

S10 Table Bacterial species related to the health states of ginseng. 0, 2, 4, 6, R2, R4 and R6 indicate non-cultivated; 2, 4, and 6 years of cultivation at first and second round (re-cultivation), respectively. JJG, Jajangri; JW, Juwolri; WD, Wondangri; JJK, Jajakri; YP, Yulpori; A, healthy soil; B, unhealthy soil. 10, 20 and 30 indicate soil samples collected from 0–10, 10–20, and 20–30 cm depths, respectively. Relative abundances are reported as the percent of total bacterial sequences observed per samples.
(XLSX)

S11 Table List of pathogenic related bacteria in ginseng soil. 0, non-cultivated; 2, 4, 6, R2, R4 and R6 indicate 2, 4, and 6 years of cultivation at first and second round (re-cultivation), respectively. JJG, Jajangri; JW, Juwolri; WD, Wondangri; JJK, Jajakri; YP, Yulpori; A, healthy soil; B, unhealthy soil. 10, 20 and 30 indicate soil samples were taken from 0–10, 10–20, and 20–30 cm in depths, respectively. Relative abundances are reported as the percent of total bacterial sequences observed per samples.
(XLSX)
