# Supplementary figures and images for: Bacterial Diversity and Community Structure in Korean Ginseng Field Soil Are Shifted by Cultivation Time
Source: PLoS One. 2016 May 17;11(5):e0155055. doi: 10.1371/journal.pone.0155055 (PMC4871511; doi:10.1371/journal.pone.0155055)

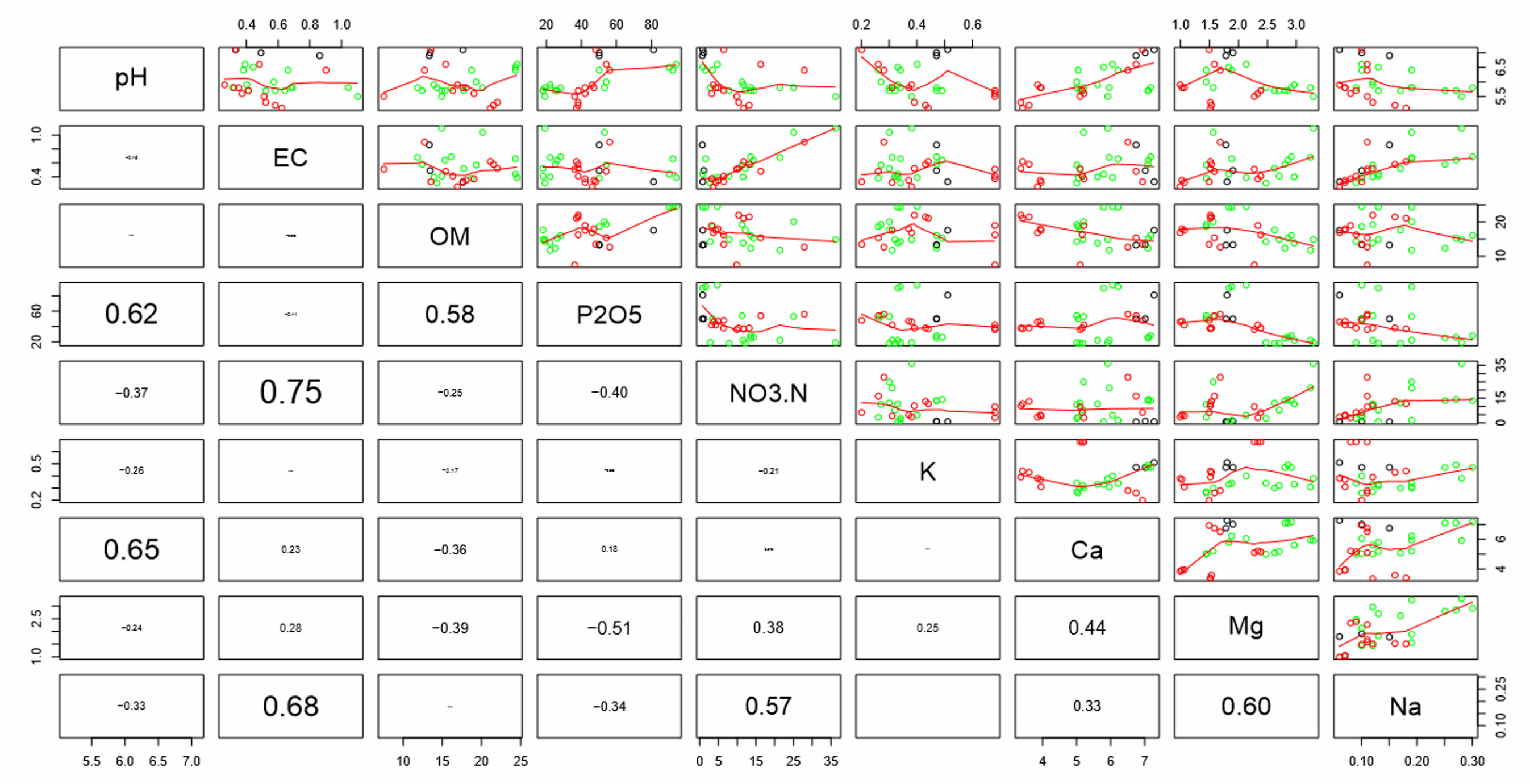

Supplement: S1 Fig — The lower diagonal panels contain the (absolute) correlation coefficients, and the upper diagonal panels contain scatterplots (and smoothing line was added). The font size of the cross-correlation is proportional to the value. Black, green and red circles indicated non-cultivation, first round and second round soil samples, respectively. (TIF) [file pone.0155055.s001.tif]
