# Supplementary material for: Bacterial Diversity and Community Structure in Korean Ginseng Field Soil Are Shifted by Cultivation Time
Source: PLoS One. 2016 May 17;11(5):e0155055. doi: 10.1371/journal.pone.0155055 (PMC4871511; doi:10.1371/journal.pone.0155055)
Supplement: S1 Table — Soil samples were collected in the city of Paju city and in Yeoncheon County (Gyeonggi-do, South Korea). (DOCX) [file pone.0155055.s002.docx]

**S1 Table**. **Description of the 30 soil samples**.

| **Sample** | **Depth (cm)** | **Coordinates** | **Areas** | **Type of soil** | **Health state of ginseng** | |
| --- | --- | --- | --- | --- | --- | --- |
| 0-JJG-10 | 0-10 | 37°57′58.2″N, 126°51′36.0″E |  | Non-cultivated | |  |
| 0-JJG-20 | 10-20 |  | Jajangri, Paju city |  |  | Healthy |
| 0-JJG-30 | 20-30 |  |  |  |  |  |
| 2-JW-A10 | 0-10 | 37°58′59.2″N, 126°54′54.7″E |  | 2 years of first cultivation | | Healthy |
| 2-JW-A20 | 10-20 |  | Juwonri,  Paju city |  |  |  |
| 2-JW-A30 | 20-30 |  |  |  |  |  |
| 4-JW-A10 | 0-10 | 37°58′54.0″N, 126°54′48.7″E |  | 4 years of first cultivation | | Healthy |
| 4-JW-A20 | 10-20 |  | Juwonri,  Paju city |  |  |  |
| 4-JW-A30 | 20-30 |  |  |  |  |  |
| 4-WD-B10 | 0-10 | 37°59′13.4″N, 126°53′00.5″E |  |  | | Unhealthy |
| 4-WD-B20 | 10-20 |  | Wondangri, Yeoncheon county | 4 years of first cultivation | |  |
| 4-WD-B30 | 20-30 |  |  |  | |  |
| 6-WD-A10 | 0-10 | 37°58′49.8″N, 126°52′42.0″E |  |  | | Healthy |
| 6-WD-A20 | 10-20 |  | Wondangri, Yeoncheon county | 6 years of first cultivation | |  |
| 6-WD-A30 | 20-30 |  |  |  | |  |
| 6-WD-B10 | 0-10 | 37°58′49.8″N, 126°52′42.0″E |  | 6 years of first cultivation | | Unhealthy |
| 6-WD-B20 | 10-20 |  | Wondangri, Yeoncheon  county |  |  |  |
| 6-WD-B30 | 20-30 |  |  |  |  |  |
| R2-JJK-A10 | 0-10 | 37°59′19.2″N, 126°51′49.8″E |  |  | | Healthy |
| R2-JJK-A20 | 10-20 |  | Jajakri, Yeoncheon county | 2 years of second cultivation | |  |
| R2-JJK-A30 | 20-30 |  |  |  | |  |
| R4-YP-A10 | 0-10 | 37°59′00.7″N, 126°57′29.4″E |  |  | | Healthy |
| R4-YP-A20 | 10-20 |  | Yulpori, Paju city | 4 years of second cultivation | |  |
| R4-YP-A30 | 20-30 |  |  |  | |  |
| R4-YP-B10 | 0-10 | 37°59′21.9″N, 126°57′46.5″E |  |  | | Unhealthy |
| R4-YP-B20 | 10-20 |  | Yulpori, Paju city | 4 years of second cultivation | |  |
| R4-YP-B30 | 20-30 |  |  |  | |  |
| R6-YP-B10 | 0-10 | 37°59′03.1″N, 126°57′22.7″E |  |  | | Unhealthy |
| R6-YP-B20 | 10-20 |  | Yulpori, Paju city | 6 years of second cultivation | |  |
| R6-YP-B30 | 20-30 |  |  |  | |  |

Soil samples were collected in the city of Paju and in Yeoncheon County (Gyeonggi-do, South Korea).
