# Supplementary material for: Bacterial Diversity and Community Structure in Korean Ginseng Field Soil Are Shifted by Cultivation Time
Source: PLoS One. 2016 May 17;11(5):e0155055. doi: 10.1371/journal.pone.0155055 (PMC4871511; doi:10.1371/journal.pone.0155055)
Supplement: S2 Table — EC, electrical conductivity; OM, organic matter. (DOCX) [file pone.0155055.s003.docx]

**S2 Table. Edaphic properties of all soil samples**.

| **Samples** | **pH**  **(1:5)** | **EC**  **(dS m^-1^)** | **OM**  **(mg kg^-1^)** | **P_2_O_5_**  **(mg kg^-1^)** | **NO_3_-N**  **(mg kg^-1^)** | **Exchangeable cations**  **(cmol^+^ kg^-1^)** | | | |
| --- | --- | --- | --- | --- | --- | --- | --- | --- | --- |
|  |  |  |  |  |  | **K^+^** | **Ca^2+^** | **Mg^2+^** | **Na^+^** |
| 0-JJG-10 | 7.1 | 0.33 | 17.6 | 81 | 0.74 | 0.51 | 7.27 | 1.8 | 0.06 |
| 0-JJG-20 | 7 | 0.49 | 13.4 | 50 | 1.01 | 0.47 | 7.01 | 1.9 | 0.1 |
| 0-JJG-30 | 6.9 | 0.86 | 13.3 | 50 | 0.65 | 0.47 | 6.74 | 1.78 | 0.15 |
| 2-JW-A10 | 6.6 | 0.39 | 24.5 | 94 | 4.63 | 0.4 | 6.05 | 2.13 | 0.1 |
| 2-JW-A20 | 6.5 | 0.44 | 24.3 | 90 | 0.74 | 0.33 | 5.79 | 1.83 | 0.13 |
| 2-JW-A30 | 6.4 | 0.66 | 24.3 | 92 | 1.52 | 0.34 | 6.21 | 1.88 | 0.19 |
| 4-JW-A10 | 5.8 | 0.69 | 16.1 | 28 | 13.5 | 0.47 | 7.17 | 2.91 | 0.3 |
| 4-JW-A20 | 5.7 | 0.64 | 15.3 | 26 | 14.3 | 0.49 | 7.1 | 2.85 | 0.27 |
| 4-JW-A30 | 5.7 | 0.58 | 12.4 | 25 | 13.54 | 0.47 | 7.09 | 2.8 | 0.25 |
| 4-WD-B10 | 5.8 | 1.04 | 20.1 | 53 | 24.97 | 0.3 | 5.2 | 1.56 | 0.19 |
| 4-WD-B20 | 6 | 0.57 | 19.3 | 54 | 11.24 | 0.27 | 5 | 1.44 | 0.12 |
| 4-WD-B30 | 6.4 | 0.38 | 18.7 | 50 | 3.4 | 0.26 | 5.04 | 1.44 | 0.1 |
| 6-WD-A10 | 5.7 | 0.52 | 17.7 | 18 | 12.11 | 0.3 | 5.09 | 2.62 | 0.17 |
| 6-WD-A20 | 5.7 | 0.41 | 14.9 | 18 | 7.77 | 0.32 | 5.18 | 2.7 | 0.13 |
| 6-WD-A30 | 5.8 | 0.31 | 14.3 | 19 | 2.77 | 0.34 | 5 | 2.47 | 0.09 |
| 6-WD-B10 | 5.5 | 1.1 | 14.9 | 19 | 36.15 | 0.38 | 5.91 | 3.29 | 0.28 |
| 6-WD-B20 | 5.8 | 0.68 | 11.8 | 22 | 21.3 | 0.31 | 5.95 | 3.24 | 0.19 |
| 6-WD-B30 | 5.9 | 0.4 | 13.9 | 22 | 11.55 | 0.33 | 5.61 | 2.96 | 0.12 |
| R2-JJK-A10 | 6.4 | 0.9 | 12.7 | 56 | 27.82 | 0.28 | 6.5 | 1.68 | 0.11 |
| R2-JJK-A20 | 6.6 | 0.48 | 15.4 | 54 | 16.22 | 0.26 | 6.74 | 1.58 | 0.11 |
| R2-JJK-A30 | 7.1 | 0.33 | 13.5 | 48 | 6.32 | 0.2 | 6.93 | 1.49 | 0.1 |
| R4-YP-A10 | 5.5 | 0.51 | 7.5 | 36 | 9.8 | 0.68 | 5.1 | 2.27 | 0.11 |
| R4-YP-A20 | 5.7 | 0.41 | 16.3 | 38 | 6.27 | 0.68 | 5.16 | 2.38 | 0.09 |
| R4-YP-A30 | 5.6 | 0.37 | 19 | 42 | 3.13 | 0.68 | 5.21 | 2.33 | 0.08 |
| R4-YP-B10 | 5.8 | 0.32 | 17.6 | 42 | 4.2 | 0.31 | 3.95 | 1.06 | 0.07 |
| R4-YP-B20 | 5.8 | 0.35 | 18 | 47 | 4.8 | 0.37 | 3.93 | 1.02 | 0.07 |
| R4-YP-B30 | 5.9 | 0.26 | 16.9 | 46 | 3.57 | 0.38 | 3.85 | 0.99 | 0.06 |
| R6-YP-B10 | 5.1 | 0.62 | 21.1 | 37 | 11.64 | 0.44 | 3.41 | 1.51 | 0.18 |
| R6-YP-B20 | 5.2 | 0.58 | 21.5 | 38 | 13.1 | 0.43 | 3.6 | 1.53 | 0.16 |
| R6-YP-B30 | 5.3 | 0.52 | 22 | 38 | 10.31 | 0.39 | 3.36 | 1.51 | 0.12 |

EC, electrical conductivity; OM, organic matter.
