# Supplementary material for: Bacterial Diversity and Community Structure in Korean Ginseng Field Soil Are Shifted by Cultivation Time
Source: PLoS One. 2016 May 17;11(5):e0155055. doi: 10.1371/journal.pone.0155055 (PMC4871511; doi:10.1371/journal.pone.0155055)
Supplement: S5 Table — EC, electrical conductivity; OM, organic matter. Bold number: p<0.05; Bold and single underline numbers p<0.01; Bold and double underline numbers p<0.001. (DOCX) [file pone.0155055.s006.docx]

**S5 Table. Spearman’s rank correlations between the relative abundances of bacterial classes and the soil chemical compositions in all soil samples**.

| **Variables** | **pH**  **(1:5)** | **EC**  **(dS m^-1^)** | **OM**  **(mg kg^-1^)** | **P_2_O_5_**  **(mg kg^-1^)** | **NO_3_-N**  **(mg kg^-1^)** | **Exchangeable cations (cmol^+^ kg^-1^)** | | | |
| --- | --- | --- | --- | --- | --- | --- | --- | --- | --- |
|  |  |  |  |  |  | **K^+^** | **Ca^2+^** | **Mg^2+^** | **Na^+^** |
| Acidobacteria | **-0.74** | -0.11 | 0.33 | -**0.42** | 0.09 | 0.12 | -**0.86** | -0.20 | -0.06 |
| Betaproteobacteria | **0.41** | -0.27 | **-0.50** | -0.05 | -0.24 | -0.13 | 0.26 | 0.27 | -0.33 |
| Anaerolineae | 0.10 | **0.39** | -0.13 | 0.28 | 0.07 | **0.43** | **0.58** | 0.13 | 0.29 |
| Alphaproteobacteria | -0.26 | -0.11 | **0.38** | 0.09 | 0.04 | -0.03 | **-0.56** | **-0.37** | -0.22 |
| Solibacteres | **-0.37** | -0.19 | 0.21 | 0.00 | -0.12 | 0.08 | **-0.66** | **-0.46** | -0.32 |
| Caldilineae | **-0.72** | **0.38** | 0.07 | **-0.43** | **0.49** | 0.34 | -0.10 | 0.33 | **0.49** |
| Gemmatimonadetes | -0.07 | -0.34 | **-0.49** | **-0.47** | -0.06 | 0.00 | -0.08 | 0.35 | -0.35 |
| Deltaproteobacteria | **0.82** | -0.16 | 0.24 | **0.77** | **-0.50** | **-0.38** | 0.25 | -**0.40** | -0.29 |
| Gammaproteobacteria | **0.61** | 0.14 | 0.06 | **0.57** | -0.21 | -0.30 | 0.36 | -0.12 | -0.04 |
| Acidobacteria_HQ645210 | 0.27 | 0.09 | -0.10 | 0.18 | 0.13 | -0.35 | 0.24 | 0.27 | 0.00 |
| Verrucomicrobiae | **0.39** | -0.02 | 0.21 | **0.49** | -0.19 | -0.19 | 0.09 | **-0.41** | -0.13 |
| Acidobacteria_EU686603 | **0.74** | -0.29 | -0.18 | **0.48** | -0.28 | **-0.46** | 0.24 | -0.35 | -**0.49** |
| Sphingobacteria | **0.81** | -0.05 | 0.16 | **0.80** | **-0.37** | -0.30 | 0.34 | **-0.38** | -0.22 |
| Nitrospira | **0.71** | -0.17 | 0.06 | **0.61** | -0.33 | **-0.54** | 0.13 | **-0.51** | **-0.36** |
| OD1_FJ547054 | **0.45** | -0.14 | 0.35 | **0.55** | -0.33 | -0.19 | 0.05 | **-0.38** | -0.18 |
| Chloracidobacterium | **0.73** | 0.00 | -0.35 | **0.36** | -0.10 | **-0.49** | **0.46** | -0.08 | -0.17 |
| Actinobacteria | 0.32 | 0.23 | -0.08 | 0.23 | 0.11 | 0.04 | **0.40** | 0.17 | 0.10 |
| Chloroflexi_GQ396871 | 0.04 | **0.37** | **-0.40** | -0.14 | 0.27 | 0.05 | **0.39** | **0.48** | 0.32 |
| Thermodesulfovibrio | **0.38** | 0.23 | **0.40** | **0.57** | -0.25 | 0.07 | **0.39** | -0.10 | 0.26 |
| Ignavibacteriae | **0.54** | 0.20 | 0.32 | **0.67** | -0.23 | -0.29 | 0.16 | **-0.38** | 0.08 |
| Phycisphaerae | **0.55** | -0.11 | 0.09 | **0.62** | -0.16 | **-0.41** | 0.11 | **-0.41** | -0.30 |
| Planctomycetacia | **0.52** | -0.18 | -0.12 | **0.40** | -0.11 | -0.30 | 0.11 | -0.32 | -0.36 |
| Clostridia | 0.19 | 0.08 | **0.37** | 0.29 | -0.01 | -0.12 | -0.01 | -0.27 | 0.14 |
| Chthonomonadetes | 0.12 | -0.17 | **-0.38** | -0.12 | -0.18 | -0.08 | -0.02 | 0.15 | -0.29 |
| Acidimicrobiia | **0.37** | -0.07 | -0.22 | 0.10 | 0.15 | -0.35 | 0.31 | 0.06 | -0.10 |
| TM7 | -0.12 | **0.48** | 0.24 | 0.03 | **0.40** | -0.19 | 0.03 | 0.19 | **0.43** |
| Rubrobacteria | -0.10 | -0.13 | -0.12 | -0.18 | 0.13 | -0.13 | -0.05 | 0.26 | -0.10 |
| Chloroflexi_HM748667 | -0.32 | -0.11 | -0.31 | **-0.38** | 0.11 | 0.24 | -0.03 | 0.30 | -0.11 |
| WS3_GU302492 | **0.78** | 0.00 | -0.08 | **0.59** | -0.28 | -0.32 | **0.43** | -0.20 | -0.18 |
| Chloroflexi_EU335161 | **0.60** | -0.13 | -0.21 | **0.42** | -0.36 | 0.04 | **0.47** | -0.13 | -0.26 |
| Acidobacteria_HM243779 | **0.40** | 0.16 | **0.39** | **0.67** | -0.28 | -0.10 | 0.17 | -0.30 | 0.08 |
| MATCR | -0.01 | 0.11 | 0.18 | 0.23 | -0.09 | -0.13 | -0.21 | -0.27 | 0.00 |
| Elusimicrobia | 0.26 | 0.12 | 0.25 | 0.33 | -0.09 | **-0.40** | -0.08 | -0.12 | 0.02 |
| Opitutae | **0.62** | 0.03 | 0.25 | **0.71** | -0.23 | -0.19 | **0.41** | -0.26 | -0.11 |
| Chloroflexi_EU133950 | **-0.47** | **0.39** | 0.03 | **-0.43** | **0.41** | 0.16 | -0.01 | **0.50** | **0.56** |
| Fimbriimonadia | **0.58** | -0.10 | 0.06 | **0.53** | -0.22 | -0.28 | 0.26 | -0.05 | -0.22 |
| Thermoleophilia | 0.02 | 0.20 | 0.18 | 0.07 | 0.17 | -0.17 | 0.07 | 0.12 | 0.17 |
| Armatimonadetes_FJ479425 | **0.39** | -0.08 | **-0.36** | 0.07 | 0.12 | **-0.50** | 0.26 | 0.10 | -0.18 |

EC, electrical conductivity; OM, organic matter. Bold number: *p*<0.05; Bold and single underline numbers *p*<0.01; Bold and double underline numbers *p*<0.001.
