# Supplementary material for: Bacterial Diversity and Community Structure in Korean Ginseng Field Soil Are Shifted by Cultivation Time
Source: PLoS One. 2016 May 17;11(5):e0155055. doi: 10.1371/journal.pone.0155055 (PMC4871511; doi:10.1371/journal.pone.0155055)
Supplement: S6 Table — The total number of reads of OTUs and taxonomic assignment are reported for each OTU. (DOCX) [file pone.0155055.s007.docx]

**S6 Table.** **Core OTUs occurring in all soil samples.**

|  | **OTU-1** | **OTU-2** | **OTU-3** |
| --- | --- | --- | --- |
| **Taxonomic assignment** | | | |
| Phylum | Acidobacteria | Acidobacteria | Proteobacteria |
| Class | Solibacteres | Solibacteres | Alphaproteobacteria |
| Order | Solibacterales | Solibacterales | Rhizobiales |
| Family | Solibacteraceae | Solibacteraceae | Bradyrhizobiaceae |
| Genus | ND | ND | Pseudolabrys |
| **Occurrence (number of reads)** | | | |
| 0-JJG-10 | 160 | 53 | 88 |
| 0-JJG-30 | 147 | 53 | 89 |
| 0-JJG-20 | 149 | 43 | 84 |
| 2-JW-A10 | 159 | 68 | 93 |
| 2-JW-A20 | 147 | 36 | 99 |
| 2-JW-A30 | 147 | 44 | 89 |
| 4-JW-A10 | 152 | 60 | 93 |
| 4-JW-A20 | 147 | 55 | 93 |
| 4-JW-A30 | 149 | 51 | 89 |
| 4-WD-B10 | 150 | 61 | 90 |
| 4-WD-B20 | 147 | 51 | 104 |
| 4-WD-B30 | 147 | 56 | 86 |
| 6-WD-A10 | 158 | 34 | 92 |
| 6-WD-A20 | 147 | 39 | 84 |
| 6-WD-A30 | 151 | 46 | 88 |
| 6-WD-B10 | 153 | 58 | 86 |
| 6-WD-B20 | 147 | 61 | 93 |
| 6-WD-B30 | 149 | 62 | 84 |
| R2-JJK-A10 | 147 | 42 | 88 |
| R2-JJK-A20 | 147 | 42 | 84 |
| R2-JJK-A30 | 149 | 40 | 91 |
| R4-YP-A10 | 147 | 46 | 92 |
| R4-YP-A20 | 147 | 47 | 87 |
| R4-YP-A30 | 147 | 43 | 84 |
| R4-YP-B10 | 149 | 57 | 96 |
| R4-YP-B20 | 147 | 79 | 95 |
| R4-YP-B30 | 149 | 55 | 94 |
| R6-YP-B10 | 151 | 34 | 117 |
| R6-YP-B20 | 147 | 58 | 92 |
| R6-YP-B30 | 151 | 46 | 93 |

The total number of reads of OTUs and taxonomic assignment are reported for each OTU.
