# Supplementary material for: Bacterial Diversity and Community Structure in Korean Ginseng Field Soil Are Shifted by Cultivation Time
Source: PLoS One. 2016 May 17;11(5):e0155055. doi: 10.1371/journal.pone.0155055 (PMC4871511; doi:10.1371/journal.pone.0155055)
Supplement: S8 Table — 0, 2, 4, 6, R2, R4 and R6 indicate 0, 2, 4, and 6 years at first and second cultivation, respectively. JJG, Jajangri; JW, Juwolri; WD, Wondangri; JJK, Jajakri; YP, Yulpori; A, healthy soil; B, unhealthy soil. (DOCX) [file pone.0155055.s009.docx]

**S8 Table**. **Rare assigned families at each site.**

| Sample | Families |
| --- | --- |
| 0-JJG | *Beutenbergiaceae, Alysiosphaera, Paludibacter, Demequinaceae, Thiobios, Luteolibacter* |
| 2-JW-A | *Tsukamurellaceae, Marivirga, Meganema, Syntrophomonadaceae, Microthrix., Bacteriovoracaceae, Rhodobacteraceae, Planktophila* |
| 4-JW-A | *Symbiobacterium, Amoebophilus, Christensenellaceae, Prochlorococcaceae* |
| 4-WD-B | Not detected |
| 6-WD-A | *Carnobacteriaceae, Ferritrophicaceae* |
| 6-WD-B | *Halomonadaceae, Epulopiscium* |
| R2-JJK-A | *Verrucomicrobiaceae, Corynebacteriaceae, Dehalococcoides, Desulfobulbaceae, Spirosoma, Oscillatoriaceae* |
| R4-YP-A | *Aeromonadaceae* |
| R4-YP-B | *Sphaerobacteraceae, Bryobacter, Patulibacteraceae* |
| R6-YP-B | *Sneathiellaceae, Thermosporotrichaceae* |
|  |  |

0, 2, 4, 6, R2, R4, and R6 indicate 0, 2, 4, and 6 years at the first and the second cultivation, respectively. JJG, Jajangri; JW, Juwolri; WD, Wondangri; JJK, Jajakri; YP, Yulpori; A, healthy soil; B, unhealthy soil.
